# Multi-isotope reconstruction of Late Pleistocene large-herbivore biogeography and mobility patterns in Central Europe

**DOI:** 10.1038/s42003-024-06233-2

**Published:** 2024-05-14

**Authors:** Phoebe Heddell-Stevens, Olaf Jöris, Kate Britton, Tim Matthies, Mary Lucas, Erin Scott, Petrus Le Roux, Harald Meller, Patrick Roberts

**Affiliations:** 1 Department of Archaeology, Max Planck Institute of Geoanthropology, Jena, Germany; 2 Institute of Oriental Studies, Indo-European Studies, Prehistoric and Early Historical Archaeology, Friedrich Schiller University, Jena, Germany; 3 Leibniz-Zentrum für Archäologie (LEIZA), MONREPOS Archaeological Research Centre and Museum for Human Behavioural Evolution, Neuwied, Germany; 4 Institue of Ancient Studies, Johannes Gutenberg University Mainz, Mainz, Germany; 5 Department of Archaeology, University of Aberdeen, Aberdeen, UK; 6 Department of Human Evolution, Max Planck Institute for Evolutionary Anthropology, Leipzig, Germany; 7 Arctic University Museum of Norway, Tromsø, Norway; 8 Department of Geosciences, University of Cape Town, Cape Town, South Africa; 9 State Office for Heritage Management and Archeology Saxony-Anhalt — State Museum of Prehistory, Halle, Germany; 10 School of Social Sciences, University of Queensland, Brisbane, Australia

**Keywords:** Stable isotope analysis, Animal migration

## Abstract

Interpretations of Late Pleistocene hominin adaptative capacities by archaeologists have focused heavily on their exploitation of certain prey and documented contemporary behaviours for these species. However, we cannot assume that animal prey-taxa ecology and ethology were the same in the past as in the present, or were constant over archaeological timescales. Sequential isotope analysis of herbivore teeth has emerged as a particularly powerful method of directly reconstructing diet, ecology and mobility patterns on sub-annual scales. Here, we apply ^87^Sr/^86^Sr isotope analysis, in combination with δ^18^O and δ^13^C isotope analysis, to sequentially sampled tooth enamel of prevalent herbivore species that populated Europe during the Last Glacial Period, including *Rangifer tarandus*, *Equus* sp. and *Mammuthus primigenius*. Our samples come from two open-air archaeological sites in Central Germany, Königsaue and Breitenbach, associated with Middle Palaeolithic and early Upper Palaeolithic cultures, respectively. We identify potential inter- and intra-species differences in range size and movement through time, contextualised through insights into diet and the wider environment. However, homogeneous bioavailable ^87^Sr/^86^Sr across large parts of the study region prevented the identification of specific migration routes. Finally, we discuss the possible influence of large-herbivore behaviour on hominin hunting decisions at the two sites.

## Abstract (web summary)

Using multi-isotope analysis of tooth enamel of reindeer, equids and mammoth from the archaeological sites of Königsaue and Breitenbach, the authors identify shifts in ranging behaviour of these hominin prey species during the Last Glacial Period in Central Germany.

## Introduction

Understanding the diets, habitat use and mobility patterns of large- herbivores can provide important insights into past ecosystem dynamics and the hunting strategies of hominins that exploited them (e.g., refs. ^1–5^). This is particularly the case in Late Pleistocene Europe where certain fauna are considered distinctive of certain environmental conditions and hominin economies between the Middle and Upper Palaeolithic (e.g., refs. ^6–10^). Traditionally, interpretations of past prey behaviour have rested on the assumption that past populations exhibited similar traits as their modern-day relatives. However, this has proven to be problematic when faced with the non-analogue environments of the Last Glacial Period in Eurasia (for an overview see ref. ^11^). The need to reconstruct ecological and ethological characteristics directly from the remains of the animals themselves has been highlighted by evidence for temporal and spatial variability in species responses to global climate fluctuations during MIS 3 (e.g., ref. ^3^). However, until recently, efforts to reconstruct the behaviours of palaeofauna at a local level on sub-annual timescales applicable to hominin decision-making have been few (but see refs. ^3,4,12–14^). Even within critical areas, such as Central Europe, this information is lacking for the most commonly occurring species in archaeological contexts, such as reindeer (*Rangifer tarandus*) and equids (*Equus* sp.), where complex cultural and environmental interactions have been predicted (see ref. ^15^ for an overview).

**Fig. 1:**
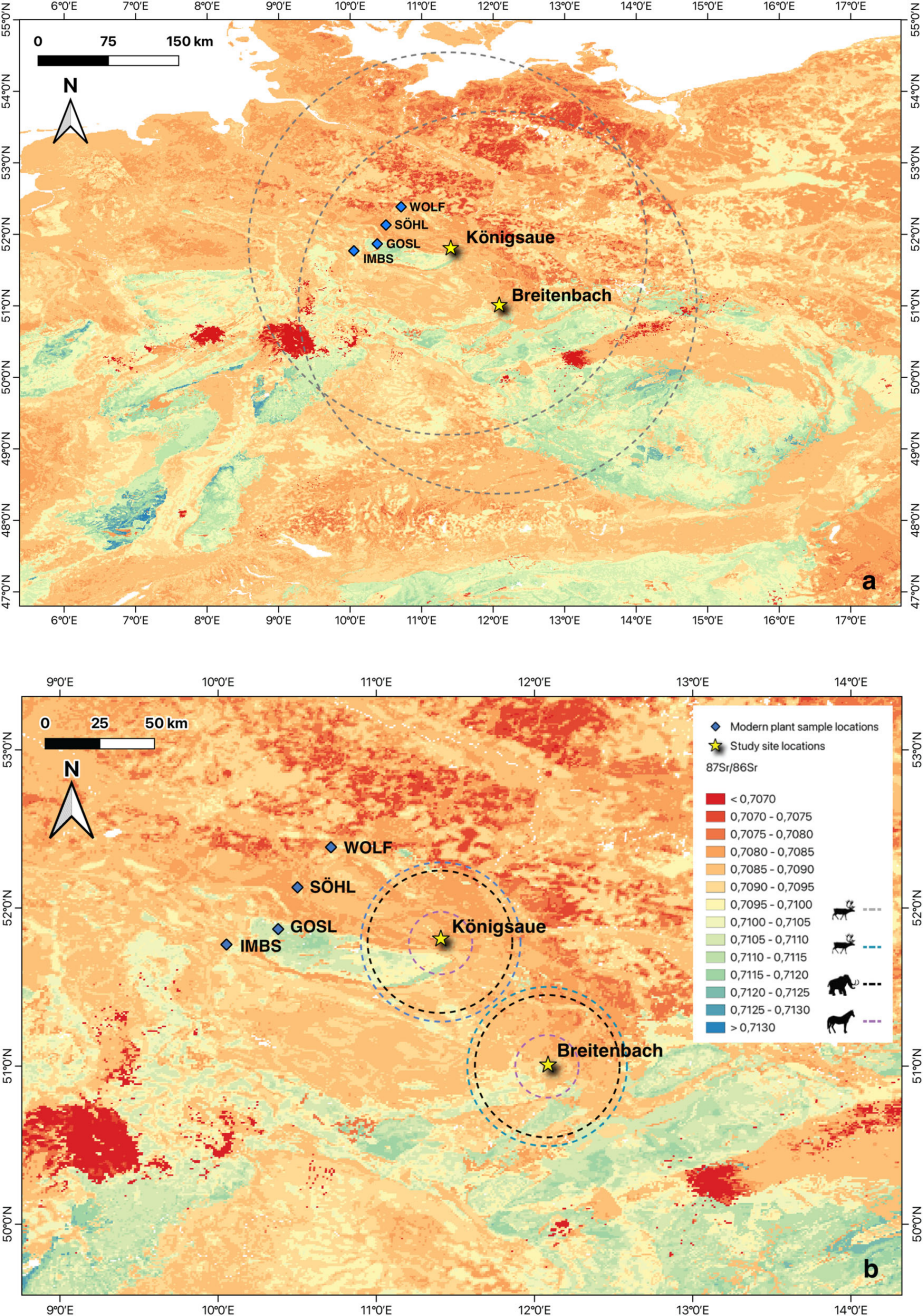
Modelled bioavailable ^87^Sr/^86^Sr isoscape from Bataille et al.^100^ of Central Europe including average home ranges sizes of extant barren-ground caribou, wild equids and African elephants and sampling locations of modern plants. **a** modelled bioavailable ^87^Sr/^86^Sr isoscape from Bataille et al.^100^ cropped to Central Europe. The grey circles represent the average distance (one-way) for spring migration/distance between winter ranges and calving grounds by migratory barren-ground caribou in Québec^32,141^. The sampling locations of the modern plant (IMBS, GOSL, SÖHL, WOLF) analysed as part of this study are marked with blue diamonds (all plant ^87^Sr/^86^Sr data provided in Supplementary Data 3). **b** modelled bioavailable ^87^Sr/^86^Sr isoscape^100^ cropped to the study area. Average home range sizes of extant sedentary woodland caribou are denoted by blue circles^29^, extant wild equids are denoted by purple circles^42^, and extant African elephant by black circles^142^.

Debates over whether or not reindeer were migratory during the European Late Pleistocene are long-standing (see refs. ^16,17^). Multiple hypotheses have been put forward, including arguments for long-distance north-south “thermo-stress” migrations (e.g., refs. ^18–20^), to smaller-scale east-west movements (e.g., refs. ^21–24^), as well as sedentary behaviours^13,17,25–28^. Extant North American caribou populations include both migratory and  resident ecotypes, and home range size within a single subspecies or herd can vary to a large degree, i.e. from a few hundred to several thousand square kilometres (Fig. 1a, b) (Supplementary Note 1)^29–32^. Seasonal movements in modern caribou depend on a range of factors including topography and plant cover^33,34^, as well as snow cover^35–37^ and predation risk^38,39^. While the number of studies undertaken is small, isotopic data from Late Pleistocene European reindeer have suggested that, although exact distances covered are difficult to infer, both migratory and non-migratory ecotypes may have been present^3,13,24^. Ecomorphological and isotope data for equids (*Equus* sp.) from Late Middle and Late Pleistocene contexts in Eurasia suggests past populations were less mobile than modern wild equids, maintaining relatively local ranges^3,14,40,41^. Maximum home range size in modern wild bands can vary from between 12 and 48.2 km^2^ in mesic steppe grasslands^42^ and forests^43,44^, to <1357 km^2^ in arid environments^45^, as movements are highly dependent on seasonal forage and water availability (Fig. 1b) (Supplementary Note 1)^42,46–48^. Evidence for migratory behaviour in the European mammoth (*Mammuthus primigenius*) during the Pleistocene is lacking, although certain individuals may have undertaken long-distance seasonal migrations^49^, similar to some modern elephant populations (Supplementary Note 1)^50,51^. Indeed, efforts to reconstruct the palaeoecology and palaeoethology of these three species are limited for Late Pleistocene contexts in Central Europe (although see refs. ^52–56^). Given the behavioural variation and plasticity seen in modern populations, obtaining this information for past animals is crucial if we are to discuss these parameters in terms of hominin adaptations^57^ and in terms of individual site-use and subsistence practices^3,58^.

**Fig. 2:**
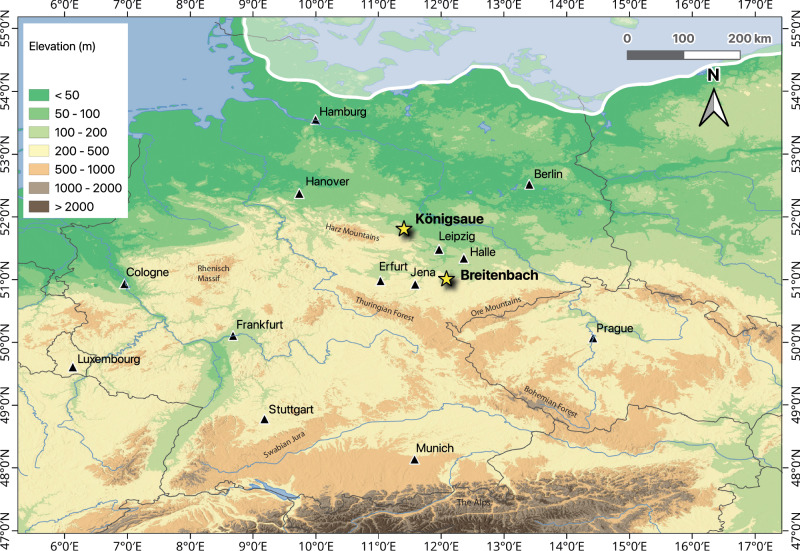
Elevation map of Central Europe including the maximum extent of the Fennoscandian ice sheet during MIS 4. Elevation map showing the locations of the study sites Königsaue (51°80’51”N, 11°40’75”E, Thuringia) and Breitenbach (51°00’78”N, 12°08’50”E, Saxony-Anhalt) (yellow stars) and major cities (black triangles). Approximate maximum extent of the Fennoscandian ice sheet within the potential period of Middle Palaeolithic hominin site occupation at Königsaue (Layer A)^90,143–148^ correlated with MIS 4 (ca. 71–57 ka BP)^71,149,150^ is shown by the thick white line. The maximum ice sheet extent during the early Upper Palaeolithic hominin occupation of Breitenbach is further north^71,150^ and therefore is not visible on the current map. Elevation map created using ETOPO1 data^151^.

While a number of detailed zooarchaeological studies of Central European Palaeolithic faunal assemblages exist^59–68^, there have been few efforts to obtain direct palaeoecological and palaeoethological data from this material (although see refs. ^55,69^). Located at the edge of the maximum southern extent of the Fennoscandian Ice Sheet during the Last Glacial Period (Fig. 2)^70,71^, Central Germany likely represented the northern periphery of hominin, and many ungulate species’ ranges during this time ^72^. There is evidence for marked climate seasonality at Central European mid-latitudes during this time^73,74^ resulting in seasonal shifts in the vegetation cover^75,76^, which are a major driver of ungulate migrations today^77,78^. If this region comprised the northern extent and coldest part of their ranges, it is likely that migratory animals inhabited this area during the warmer months, moving south or to the west for the colder part of the year, thereby avoiding seasonal climate extremes. As such, this geographic context presents an important opportunity to investigate mobility patterns in ungulates during the Last Glacial Period^24^. Multi-isotope analysis of faunal remains from archaeological sites is an established method of reconstructing past animal behaviour directly from the remains themselves (Supplementary Note 2) (e.g., refs. ^38–40^). Strontium isotope (^87^Sr/^86^Sr) analysis is increasingly employed in mobility studies of modern and ancient terrestrial animals^3,12,79–81^ due to ^87^Sr/^86^Sr values in tooth enamel being largely derived from those of the local lithologies occupied by that individual during the period of enamel formation (Supplementary Note 3, Supplementary Table 1)^82,83^. Stable oxygen isotope (δ^18^O) analysis provides insights into water ingested by an animal which, in turn, reflects δ^18^O drinking water sources often fed by local precipitation and plant water derived from environmental water^84,85^. At northern hemisphere mid-latitudes δ^18^O exhibits seasonal variation, with decreases in intra-tooth δ^18^O values reflecting cooler, wetter winter climate conditions while increases reflect summer conditions^86–88^. Additionally, shifts in intra-tooth stable carbon isotope (δ^13^C) data, due to plant type and degree of environmental closure, can provide an additional line of evidence for individual mobility patterns by revealing changes in diet related to movement between habitats^4,14,89^.

Here, we analyse ^87^Sr/^86^Sr isotope ratios of enamel bioapatite and δ^18^O and δ^13^C values of enamel carbonate of sequentially-sampled herbivore teeth from two open-air sites, Königsaue and Breitenbach, from Central Germany, in order to reconstruct the seasonal dietary and ranging habits of predominant prey-taxa. Königsaue Layer A (KÖA)^90,91^ has produced Middle Palaeolithic cultural material associated with Neanderthals, while Breitenbach (BRE)^92,93^, dates to the early Upper Palaeolithic^94,95^ and is associated with modern human Aurignacian artefacts (site backgrounds in Supplementary Note 4) (Fig. 1). We selected the most commonly represented herbivore species from each site (based on taxonomic counts), namely reindeer and horse, in addition to a mammoth individual from KÖA (Supplementary Tables 2–3). One of the challenges of utilising strontium analysis for mobility studies, particularly in Pleistocene Eurasia, is the movement of sediments by wind, water or glacier action^96^ which can obscure ^87^Sr/^86^Sr ratios of local bedrock, necessitating the development of bioavailable ^87^Sr/^86^Sr baselines and multi-isotope approaches (e.g., refs. ^97–99^). In this study, we interpret individual animal movements (intra-tooth ^87^Sr/^86^Sr data) in terms of the bioavailable environmental ^87^Sr/^86^Sr isoscape produced by Bataille et al.^100^ (Fig. 2). We identify inter-site differences in ranges and mobility patterns for different species. While we were unable to identify specific migration routes of individuals, largely due to the homogeneity of bioavailable ^87^Sr/^86^Sr values in the study region, we could rule out movement into certain areas during enamel mineralisation, and determine the potential for long-distance migrations, possibly on an east-west axis. Our study highlights the importance of multi-isotope approaches to reconstructing past large-herbivore behaviour and the potential for this information to inform on past hominin hunting decisions.

## Results

### Bioavailable environmental strontium (^87^Sr/^86^Sr)

Grass, shrub and tree leaf samples were collected from four locations along a ~90 km long southwest-northeast transect on the northern edge of the Thuringian Basin (Fig. 1). We collected and analysed three plant samples (grass, shrub and tree) from each of the four sample locations (IMBS, GOSL, SÖHL, WOLF) (Fig. 1). The plant material from the sample sites displays mean ^87^Sr/^86^Sr values of 0.7101 ± 0.000012 (IMBS), 0.7139 ± 0.000019 (GOSL), 0.7094 ± 0.000012 (SÖHL), 0.7114 ± 0.000019 (WOLF) from south to north respectively (Supplementary Data 3). These values correlate closely with modelled bioavailable ^87^Sr/^86^Sr for these areas^100^ (Fig. 1).

### Faunal intra-tooth isotope data

**Fig. 3:**
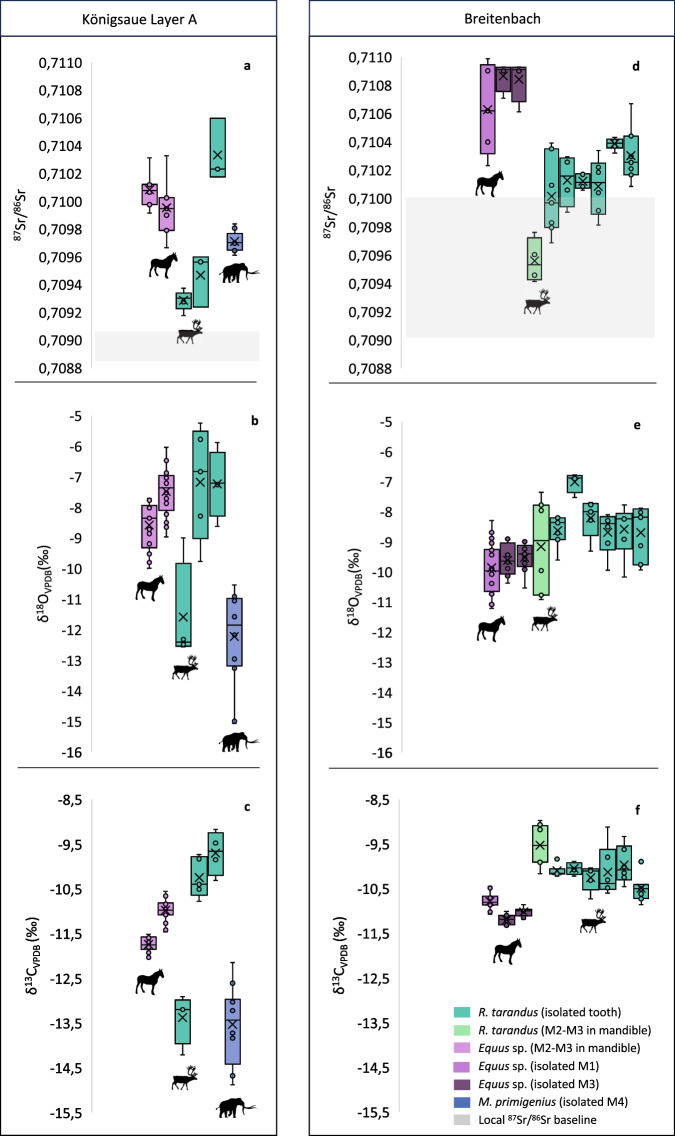
Box and whisker plots showing the range and distribution of intra-tooth ^87^Sr/^86^Sr, δ^18^O, and δ^13^C data for individual horses, reindeer and mammoth from Königsaue Layer A and Breitenbach. **a** combined intra-tooth ^87^Sr/^86^Sr values, **b** intra-tooth δ^18^O values, and **c** combined intra-tooth δ^13^C values for *Equus* sp. (*n* = 2), *R. tarandus* (*n* = 3), and *M. primigenius* (*n* = 1) individuals from KÖA. **d** combined intra-tooth ^87^Sr/^86^Sr values, **e** combined intra-tooth δ^18^O values, and **f** combined intra-tooth δ^13^C values for *Equus* sp. (*n* = 3) and *R. tarandus* (*n* = 7) individuals from BRE. Each box represents one individual. Light pink boxes represent equid M2-M3 teeth in the mandible, light purple boxes represent the isolated M1 equid tooth, dark purple boxes represent isolated M3 equid teeth. Dark teal boxes denote isolated reindeer teeth (all M3) and light teal boxes represent reindeer M2-M3 teeth in the mandible. The blue box represents the single isolated mammoth tooth (M4). All numerical source data is provided in Supplementary Data 1–2. Silhouettes sourced from PhyloPic^152^ under Creative Commons license (CC0 1.0 Universal Public Domain Dedication).

**Fig. 4:**
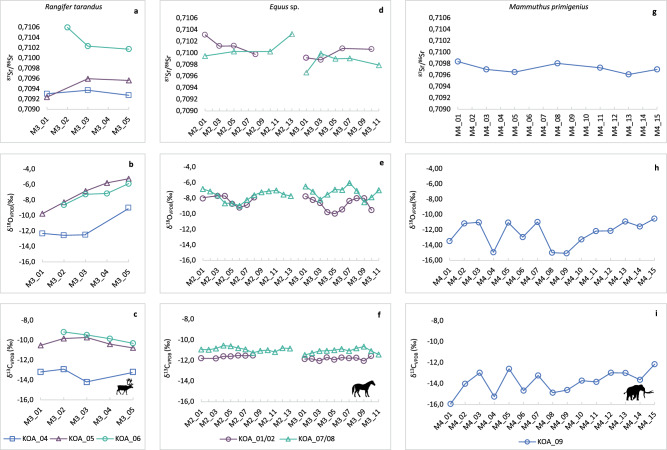
Intra-tooth enamel isotope data for *Rangifer tarandus*, *Equus* sp. and *Mammuthus primigenius* individuals from Königsaue Layer A. **a** ^87^Sr/^86^Sr, **b** δ^18^O, and **c** δ^13^C intra-tooth (isolated M3) values from *R. tarandus* (*n* = 3, KÖA_04-06). **d** ^87^Sr/^86^Sr, **e** δ^18^O, and **f** δ^13^C intra-tooth (M2-M3 in mandible) values from *Equus* sp. (*n* = 2, KÖA_01/02, KÖA_07/08). **g** ^87^Sr/^86^Sr, **h** δ^18^O, and **i**, δ^13^C intra-tooth (isolated M4) values from *M. primigenius* (*n* = 1, KÖA_09). Individual sample positions along the tooth crown are plotted from the occlusal surface to the enamel root junction (left to right), with higher sample numbers representing earlier forming enamel. Each individual is represented by a different colour/symbol. Isotope measurements are listed in full in Supplementary Data 1. Silhouettes sourced from PhyloPic^152^ under Creative Commons license (CC0 1.0 Universal Public Domain Dedication).

**Fig. 5:**
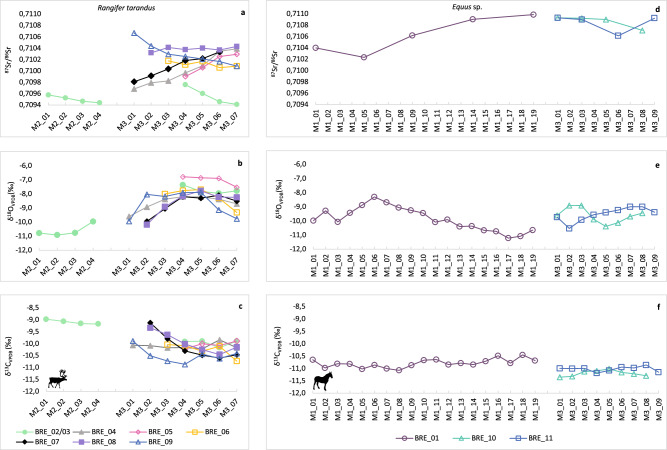
Intra-tooth enamel isotope data for *Rangifer tarandus* and *Equus* sp. individuals from Breitenbach. **a** ^87^Sr/^86^Sr, **b** δ^18^O, and **c** δ^13^C intra-tooth values of M2-M3 in mandible (BRE_02/03) and isolated M3 teeth (BRE_04-09) from *R. tarandus* (*n* = 7). **d** ^87^Sr/^86^Sr, **e** δ^18^O, and **f** δ^13^C intra-tooth values of isolated M1 (BRE_01) and M3 (BRE_10, BRE_11) teeth from *Equus* sp. (*n* = 3). Individual sample positions along the tooth crown are plotted from the occlusal surface to the enamel root junction (left to right), with higher sample numbers representing earlier forming enamel. Each individual is represented by a different colour/symbol. All isotope measurements are listed in full in Supplementary Data 2. Silhouettes sourced from PhyloPic^152^ under Creative Commons license (CC0 1.0 Universal Public Domain Dedication).

All isotope measurements are listed in full in Supplementary Data 1–2. We analysed 254 enamel samples from (*n* = 19) teeth belonging to *R. tarandus* (*n* = 10), *Equus* sp. (*n* = 5) and *M. primigenius* (*n* = 1) individuals across the two study sites. The individual range of intra-tooth δ^13^C, δ^18^O and ^87^Sr/^86^Sr values for each animal by species and site are presented in Fig. 3a–f. The intra-tooth δ^13^C, δ^18^O and ^87^Sr/^86^Sr data for each animal is presented in Figs. 4–5.

### Königsaue Layer A (KÖA)

Three permanent reindeer third molars (M3) were analysed from the KÖA assemblage representing approximately 6 months of life, taking into account enamel wear (Fig. 4a–c) (Supplementary Note 3)^101^. Combined intra-tooth ^87^Sr/^86^Sr values range between 0.7092 and 0.7106 (Fig. 4a). Reindeer display the largest inter-individual range in strontium values of the three species analysed from the site (Fig. 3a). KÖA_04 and KÖA_05 ^87^Sr/^86^Sr values fall within the modelled range of bioavailable ^87^Sr/^86^Sr (0.7082–0.7097) for the site environment. These two individuals display overlapping intra-tooth ^87^Sr/^86^Sr values closest to the occlusal surface (OS), suggestive of spatial proximity during enamel mineralisation. In contrast, KÖA_06 exhibits consistently higher intra-tooth ^87^Sr/^86^Sr values (0.7102–0.7106) indicative of occupying a different lithology. Reindeer δ^18^O values range from −12.6 to −5.3‰ (Fig. 4b). Across the three reindeer there is an increase in intra-tooth δ^18^O values between the M3 OS and enamel root junction (ERJ). This increase along the tooth crown likely reflects spring-summer climate conditions in line with the predicted timing of enamel mineralisation based on an early June birth season^24,79^. While δ^18^O values of KÖA_04 and KÖA_05 overlap, those of KÖA_06 intra-tooth are ~2‰ lower. The same pattern is seen in the intra-tooth δ^13^C values (Fig. 4c). The combined range of δ^13^C values for the reindeer is −14.2 to −9.2‰ indicative of some browse and/or shrub consumption at the lower end (KÖA_06) and grazing in more open environments at the higher end (KÖA_06) during this period.

The two equid individuals analysed from KÖA exhibit M2-M3 intra-tooth ^87^Sr/^86^Sr values between 0.7097 and 0.7103, corresponding to a combined ~3.5-year period of enamel mineralisation (Supplementary Note 3) (Fig. 4d)^102^. These values are in the upper range of the modelled site baseline and exhibit less inter-individual variation in comparison to the KÖA reindeer. While both equids exhibit overlap between M2-M3 ^87^Sr/^86^Sr values, there is a similar trend towards higher values in the M2 teeth. The two horses exhibit a similar semi-sinusoidal pattern and range in M2-M3 intra-tooth δ^18^O, likely reflecting seasonal variation in local δ^18^O of precipitation-fed drinking water sources (Fig. 4e)^89,103^. Intra-tooth δ^13^C values range between −11.5 and −10.6‰ for both horses, indicative of predominantly grazing in more open environments with very little change in diet indicated during enamel mineralisation (Fig. 4f). The equids exhibit the smallest range in δ^13^C values of the three species sampled from KÖA.

An M4 from a single mammoth was also analysed from KÖA (Fig. 4g–i). Taking into account enamel wear, the sampled enamel is estimated to represent a total of ~4 years of life (Supplementary Note 3)^104,105^. Intra-lamella ^87^Sr/^86^Sr values range from 0.7096 to 0.7099 (Fig. 4g) and display ~0.0002 annual-scale shifts. Intra-lamella δ^18^O values range between −15 and −10.5‰ (Fig. 4h). The mammoth δ^18^O values exhibit a roughly semi-sinusoidal pattern in the first half of the crown before steadily increasing towards the ERJ. As presumed obligate drinkers^106,107^, the trend in the first half of the tooth may reflect seasonal variation in δ^18^O of local precipitation-fed drinking water, while the pattern in the second half of the crown may be indicative of a change to a seasonally-buffered drinking water source. Intra-lamella δ^13^C values (−15.9 to −12.2‰) are indicative of mixed-feeding, with lower intra-tooth values (<14‰) indicative of greater browse intake and feeding in forest or woodland habitats^104,105^, while roughly parallel trends in intra-tooth δ^13^C and δ^18^O are suggestive of increased browse consumption during the colder months (Fig. 4i).

### Breitenbach (BRE)

From the Breitenbach reindeer material, six isolated M3 teeth (BRE_04 to 09), and an M2-M3 in mandible (BRE_02/03) were analysed (Fig. 5). The predicted combined period of enamel formation for M2 and M3 teeth is ~15 consecutive months without wear, beginning during the first summer of an individual’s life (Supplementary Note 3)^24,79,101^. The combined intra-tooth ^87^Sr/^86^Sr range for the Breitenbach reindeer is 0.7094–0.7107 (Fig. 5a). Inter-individual variation in ^87^Sr/^86^Sr values is greatest in the sample positions closest to the OS in M3 teeth, while the least variation is seen in those closest to the M3 ERJ. The combined M2-M3 intra-tooth δ^18^O values of the reindeer range from −10.9 to −6.8‰ and display a semi-sinusoidal pattern indicative of seasonal variation in δ^18^O of total ingested water (Fig. 5b). All reindeer intra-tooth δ^18^O values peak around the M3 mid-crown sample position, likely correlating with the summer months. Intra-tooth δ^13^C values in the reindeer enamel exhibit a range of −10.9 to −9‰ (Fig. 5c). The δ^13^C values in the M3 teeth are lower, suggestive of grazing in open environments and a decrease in lichen intake during the warmer months. Inter-individual variation in δ^13^C values is reduced in the second half of the M3 crown, indicative of more similar diet composition among reindeer during this time. The single M2 tooth analysed (BRE_02/03) displays the highest δ^13^C values of the sampled reindeer from the site, likely reflecting increased lichen consumption during the colder months, a behaviour widely observed in modern caribou^108^.

A single M1 tooth (BRE_01) and two M3 teeth (BRE_10, BRE_11) from three equid individuals were analysed from Breitenbach (Fig. 5d–f). These animals exhibit an intra-tooth ^87^Sr/^86^Sr range of 0.7102–0.7110 (Fig. 4d). These values are at the upper limit of the predicted range for the site area and are higher than the majority of those seen in the Breitenbach reindeer. BRE_01 (M1) exhibits the greatest intra-tooth range in ^87^Sr/^86^Sr values (0,0008), the lowest of which overlap with the highest ^87^Sr/^86^Sr values of the reindeer. BRE_10 and BRE_11 exhibit smaller intra-tooth ^87^Sr/^86^Sr ranges (0.0002 and 0.0003 respectively), at the higher end of those of BRE_01. The Breitenbach equid intra-tooth δ^18^O values range between −11.4 and −10.5‰ (Fig. 5e). BRE_10 and BRE_11 exhibit a semi-sinusoidal pattern in intra-tooth δ^18^O, likely reflective of seasonal variation in δ^18^O of local precipitation-fed sources of drinking water. Intra-tooth δ^13^C values of the three equids display a range of −11.4 to −10.5‰, largely indicating of grazing with little variation in diet during enamel mineralisation (Fig. 5f).

## Discussion

In this study, we aimed to interpret faunal ^87^Sr/^86^Sr data in terms of spatial distribution and seasonal movement by individuals across the landscape. However, there are difficulties in using ^87^Sr/^86^Sr values to identify movements of large herbivores during the Late Pleistocene in this region, and indeed more widely in Western and Central Europe^24^, as a result of an overall geological homogeneity particularly on the North European Plain^96,109,110^. Modelled bioavailable ^87^Sr/^86^Sr is similar across much of Central Germany (0.7085–0.7095), including the study area, as well as large areas of Northwestern and Western Europe extending into Belgium and the Netherlands, which are interspersed with smaller areas of lower (0.7075–0.7085) and higher values (0.7095–0.7100) (Fig. 2)^100^. The majority of the ^87^Sr/^86^Sr values of the animals sampled in this study fall within this range and therefore these individuals could have plausibly undertaken long-distance east-west movements within this region without large-scale shifts in enamel ^87^Sr/^86^Sr. However, some smaller isotopically distinct regions can be identified, in particular, an area of predominantly lower ^87^Sr/^86^Sr values (<0.7070–0.7075) that extends from the Northeastern edge of the study area across the North European Plain to the Baltic coast. Furthermore, in elevated areas, including the Harz, Slate, and Ore Mountains, and the Thuringian Forest range, a positive relationship can be identified between increasing altitude and bioavailable strontium values. The ^87^Sr/^86^Sr values of foothills and lower slopes range between ~0.7100–0.7105 and the values reach >0.7130 on mountain tops. As none of the individuals in this study display ^87^Sr/^86^Sr values below 0.7090 or above 0.7110, we suggest that it is therefore unlikely that these animals spent significant time to the north of the study area towards the coast or at higher elevation during the period of enamel mineralisation.

Due to these difficulties in assigning animal ranges to specific locations we focus the remainder of our discussion on the intra- and inter-site site differences in seasonal mobility patterns of reindeer, horse and mammoth. The warm season biogeography of the three Königsaue Layer A reindeer displays potential differences in spring-summer range and mobility patterns. A lack of overlap in ^87^Sr/^86^Sr values with the other individuals suggests that reindeer KÖA_04 moved over different lithologies during these months, and may have spent the spring period in a slightly elevated area (i.e., low hills or foothills) (Fig. 4a)^38,111^. Furthermore, inter-individual differences in δ^18^O and δ^13^C values indicate differences in dietary habits and environmental conditions between the KÖA reindeer (Fig. 4b, c). This may be the result of differences in home range and mobility patterns (including more or less mobile animals) among individuals from the same herd during the same year, inter-annual differences between a herd, or the presence of different herds/populations at the site. The Breitenbach reindeer exhibit higher ^87^Sr/^86^Sr values in general than those of the KÖA individuals (Fig. 3a, d). The greatest differences in spatial distribution between the BRE reindeer appear to have been during spring (Fig. 5a). Individual ranges display increasing overlap during summer and autumn, indicating that these animals may have occupied similar lithologies during this time. This inter-individual trend in ^87^Sr/^86^Sr values is mirrored by a parallel trend in δ^13^C values (Fig. 5a, c). It appears the BRE reindeer also experienced comparable seasonal variation in environmental δ^18^O during the spring-autumn months, further suggestive of similar ranges (Fig. 5b)^84,85^. Similarities in autumn ranges of reindeer at Breitenbach likely reflects these individuals assembling at the end of summer for the rut and autumn migration, akin to modern *R. tarandus* (Supplementary Note 1). Furthermore, the position of the site, directly adjacent to a Late Pleistocene river channel, may reflect the use of this part of the waterway as a crossing point for reindeer, concentrating the herd during migration^112^. If larger-scale movements were undertaken by the KÖA and BRE reindeer it appears most likely that they would have been along a predominantly east-west axis. East-west migrations have previously been argued for reindeer populations in Northern Germany at the Final Upper Palaeolithic site of Stellmoor^24^. On the basis of intra-tooth isotope data, Price et al.^24^ suggest the site was located close to the reindeers’ winter range with summer grazing areas to the east, which is supported by zooarchaeological evidence for autumn hunting at Stellmoor^24^.

The two horses from KÖA appear to have undertaken movements across similar lithologies during the period of enamel mineralisation (Fig. 4d). Small-scale shifts in intra-tooth ^87^Sr/^86^Sr values may reflect seasonal movements between habitats, supported by a seasonal signal in forage intake in KÖA_07/08. The equids exhibit higher strontium values than two of the three reindeer from the site, in the range of those modelled for foothills in the region, the closest being the Harz Mountains to the southeast. Close similarities in intra-tooth δ^18^O values in these horses point to similar drinking water sources, while the strong seasonal trends are indicative of these individuals remaining at one locality during enamel mineralisation (Fig. 4e)^113,114^. Evidence for more sedentary behaviour in the KÖA horses is also supported by their δ^13^C values, indicative of year-round grazing open environments (Fig. 4f). In combination, these isotope data indicate these animals had similar dietary habits and likely occupied similar local ranges, with small-scale movements probably correlating with seasonal changes in the local environment. The BRE equids also exhibit generally higher ^87^Sr/^86^Sr values compared to reindeer at the site, consistent with those found in the vicinity of the site and various foothills to the south (i.e. the Thuringian Forest, Slate Mountains, or Ore Mountains) (Fig. 5d). Intra-tooth trends in δ^18^O values of equids BRE_10 and BRE_11 appear reflective of seasonal variation in δ^18^O of drinking water sources (Fig. 5e). A loose correlation between trends in ^87^Sr/^86^Sr and δ^18^O values of these individuals is suggestive of seasonal movements, further supported by the timing of small-scale intra-annual shifts in diet. In comparison, equid BRE_01 may have been slightly wider ranging, as indicated by comparable trends in intra-tooth ^87^Sr/^86^Sr and δ^18^O values, the latter pointing to a potential change in different drinking water sources during enamel mineralisation. Our findings correlate with those of other studies of Late Pleistocene equids from European contexts^3,14,115^ in that we find no clear evidence for large-scale movements by horses at either site. Range size in extant wild horses can vary extensively^46,116^, and is understood as being largely dependent on forage and water availability^46,48,117^. This would explain the suggested seasonal movements of the KÖA horses, which have also been hinted at for equids during the Late Glacial period in Northern Italy^14^.

The mammoth from KÖA displays regular undulations in the intra-tooth ^87^Sr/^86^Sr value implying regularity of movement over marginally different lithologies on an annual basis (Fig. 4g). Meanwhile, the relatively large-magnitude seasonal-scale variation seen in the intra-tooth δ^18^O values of this individual may be indicative of east-west movements, potentially reflecting increasing ^18^O enrichment that is seen in modern precipitation from west to east across Central Europe (Fig. 4h)^118^. Alternatively, potentially, this trend may suggest this animal spent time in an area of greater continentality, i.e. Eastern Europe, during the period of enamel mineralisation. The seasonal-scale variation in intra-tooth δ^18^O values is mirrored in the δ^13^C values, and are indicative of strong warm-cold season differences in feeding behaviours that include grazing in the summer months and shift to browsing and/or feeding in more closed environments in the winter (Fig. 4i). This trend towards greater grass consumption during the warmer months is also seen in Late Glacial mammoths in North America^119^. The close correlation between δ^18^O and δ^13^C may also point to the strong influence of dietary δ^18^O in the enamel oxygen isotope signal. For example, leaf water in grasses generally displays stronger leaf water enrichment compared to source water and to leafy dicotyledonous plants^113,120,121^, while deep-rooted trees with access to subsurface waters are more likely to exhibit leaf water δ^18^O values closely related to those of local precipitation^122,123^. These trends may therefore compound variation in the mammoth intra-tooth δ^18^O values produced by seasonal fluctuations in δ^18^O of meteoric drinking water. The δ^13^C values of the KÖA mammoth are lower than those previously obtained from mammoth enamel carbonate within European contexts during the Last Glacial Period, which display a trend towards lower δ^13^C values in Northeastern Europe^49^, suggesting that this individual might also have spent time in this area. In summary, it is possible that the KÖA mammoth moved over longer distances across similar lithologies (or at least lithologies resulting in similar bioavailable ^87^Sr/^86^Sr) in an east-west direction. Regardless of scale, mobility patterns in ancient proboscideans, like modern elephants^124,125^, are assumed to have been predominantly driven by food availability^126^, which may also explain the movements of the KÖA individual. Although the specifics of European mammoth migration remain unclear^126^, recent evidence suggests large-scale north-south seasonal movements were undertaken by an individual discovered in the Polish Gravettian context^49^.

Despite issues with the ^87^Sr/^86^Sr baseline homogeneity in the region, our multi-isotope work has enabled us to reconstruct different behaviours and ecologies in these three important large-herbivore species in Late Pleistocene Central Europe. Furthermore, comparing faunal intra-tooth isotope data with zooarchaeological and material culture data potentially yields evidence for differences in hominin hunting seasonality and practices at Königsaue Layer A and Breitenbach. Currently, season-of-death data is only available for reindeer from the two sites. From KÖA, very few cut-marked bones have been identified, and the location of the site on an ancient lakeshore increases the potential for the natural accumulation of animals. Zooarchaeological evidence indicates that reindeer (*n* = 3) died between summer and autumn (Supplementary Note 4)^90^. The multi-isotope results presented here suggest reindeer individuals could have been in the vicinity of Königsaue during summer and potentially spring, but could also have been elsewhere on comparable lithologies. To date, season-of-death has been determined as late autumn-early winter for a single reindeer individual from Breitenbach (Supplementary Note 4)^127^. An autumn migration route for reindeer in the site vicinity has potential support from the ^87^Sr/^86^Sr data obtained in this study. But perhaps more informative is the behavioural similarities between the different individuals analysed in terms of their seasonal biogeography, particularly in autumn, as indicated by the strontium isotope results. While these animals could have belonged to one or more herds, these results suggest that the BRE reindeer exhibited a more uniform spatial distribution and were potentially concentrated in an isotopically similar area during autumn in the area of the site. This may therefore be the result of reindeer passing close to the site during their autumn migration. Fidelity to the autumn range and migration route would have made reindeer a predictable (in terms of location) and potentially more attractive (in terms of location and numbers) to human hunters. Furthermore, inter-individual similarities in isotope signals may also support an anthropogenic explanation for the accumulation of the BRE reindeer material (i.e. that they were hunted during a single or closely related event/s). One interpretation might be that while the KÖA assemblage could represent encounter hunting in the lakeside environment, the BRE assemblage may be indicative of interception hunting of reindeer during autumn migration, potentially where they crossed the adjacent river. This hunting strategy has been well documented in historical accounts which describe hunters targeting reindeer as they aggregated to traverse rivers and lakes as they are slower in the water^112,128^, and has also been argued for archaeologically for both Neanderthals and modern humans^129–132^.

The data presented here provide novel evidence for the biogeography of three different Late Pleistocene large-herbivore species and the ways these behaviours may have intersected with their exploitation by hominin hunters. However, while it is possible to elucidate relative intra- and inter-species differences and commonalities – and to observe intra-tooth variation consistent with seasonally-varied range use (i.e., migrations) – it is not possible to firmly identify specific geographical animal ranges and migration routes. This problem could, in part, be overcome with increased (and higher density) local sampling for bioavailable ^87^Sr/^86^Sr in Central Europe in order to establish higher resolution baselines. However, given the homogeneity in lithology and surficial deposits over large swathes of the region, the incorporation of other isotope analyses, such as sulphur (δ^34^S), should be considered in future studies as an additional line of evidence for herbivore palaeomobility. However, the current data presented here are sufficient to yield context-specific insights into the relationship between hominin hunting strategies and contemporary animal behaviour, insights that take into account locally specific ecological differences. In so doing, our study further demonstrates the importance of reconstructing sub-annual mobility patterns and dietary habits of hominin prey-taxa through the use of direct methods applied to the remains of animals from archaeological sites^3,13,133^.

## Methods

### Sampling of faunal tooth enamel

Six teeth belonging to five individuals from Königsaue Layer A were selected on the basis of availability including three permanent lower third molars from *Rangifer tarandus*, two permanent lower second and third molars from *Equus* sp., and one permanent first molar from *Mammuthus primigenius*. From Breitenbach we selected eight teeth from seven *R. tarandus* individuals including one permanent lower second molar and six permanent lower third molars in addition to three permanent lower third molars from three *Equus* sp. individuals. We preferentially selected later-forming teeth to avoid the weaning effect in cervids and equids (Supplementary Note 3), and those from the same side (lingual or buccal) in order to avoid sampling the sample individual twice. Identifications were carried out using the modern reference collections of the Max Planck Institute of Geoanthropology, Jena, Germany and the MONREPOS Archaeological Research Centre and Museum for Human Behavioural Evolution, Neuwied, Germany, in combination with published guides^134^. The Königsaue archaeological material is stored in the State Museum of Prehistory, Halle, Germany while the material from Breitenbach is temporarily being housed at the MONREPOS Archaeological Research Centre and Museum for Human Behavioural Evolution, Neuwied. Access to these collections may be obtained via application to the State Museum of Prehistory, Halle, Germany.

Each tooth was cleaned to remove any dirt from the burial environment using a Dremel drill and dental calculus was removed from the sampling surface. Sequential samples of powdered enamel were obtained using a handheld diamond-tipped Dremel drill along the length of the tooth crown in the growth direction (i.e., from cusp to cervix) from ~2 mm below the occlusal surface to ~2 mm above the enamel root junction. For stable carbon and oxygen isotope analysis, ~10 mg of enamel powder was taken for analysis. This amount was determined based on the 3–3.5 mg needed for analysis with a Thermo GasBench 2 connected to a Thermo Delta V Advantage Mass Spectrometer (Thermo Fisher Scientific), potential loss of sample during pre-treatment (<2 mg), and to allow for the sample to be analysed again if necessary. Individual samples of ~3 mm in breadth were obtained in parallel to the growth axis. From the equid molars, sequential samples were obtained at ~3 mm intervals. From the reindeer molars, we took sequential samples as close to the previous sample as possible. Sequential samples were also taken from a single mammoth lamella (M4) at intervals of ~4–5 mm. This sampling strategy was based on the specific timing of enamel mineralisation of each tooth (Supplementary Note 3) in order to best capture seasonal-scale variation in the enamel isotope signal^24,79,102^. For strontium isotope analysis, we removed an additional ~15 mg of enamel powder from each of the *Rangifer* intra-tooth sample positions. From the *Equus* sp. molars and *Mammuthus* lamella, we took additional (~15 mg) enamel powder for ^87^Sr/^86^Sr isotope analysis from the tooth cusp and cervical sample positions and at regular increments in between relating to approximately 6-month intervals based on the timing of enamel mineralisation for each tooth. Finally, we took bulk enamel samples from the length of the tooth crown from ~2 mm below the occlusal surface to ~2 mm above the enamel root junction. We obtained a total of 254 faunal tooth enamel samples, 165 of which were analysed for stable carbon and oxygen isotope analysis and 89 of which were selected for strontium isotope analysis.

### Stable carbon and oxygen isotope analysis of faunal tooth enamel

The stable carbon and oxygen isotope analysis of faunal tooth enamel was performed at the Stable Isotope laboratory at the Max Planck Institute of Geoanthropology, Jena, Germany. Prior to analysis, the powdered enamel samples underwent a wet chemistry pre-treatment to remove organic or secondary carbonate contaminates. This consisted of a wash in 1 ml of 1% bleach solution (NaOCl) for 60 min followed by three rinses in purified H_2_O and centrifugation. After which, 1 mL of 0.1 M acetic acid was added to each sample for 10 min, followed by three more rinses in purified H_2_O and centrifuging (as per Sponheimer^135^ and Lee-Thorp et al.^136^). Samples were freeze-dried overnight before 3–3.5 mg was weighed out for analysis. Following the reaction with 100% phosphoric acid, gases evolved from the samples were analysed to establish their stable carbon and oxygen isotopic composition using a Thermo GasBench 2 connected to a Thermo Delta V Advantage Mass Spectrometer (Thermo Fisher Scientific). Stable carbon and oxygen isotope values were then compared against the international standard (NBS 18) registered by the International Atomic Energy Agency. Results are reported as delta (δ) values as parts per thousand (per mil, ‰) relative to the international standard Vienna Pee Dee Belemnite for ^13^C/^12^C and ^18^O/^16^O. Where δ(‰) = ((R_sample_/R_standard_) − 1) × 1000, and R is the ^13^C/^12^C or ^18^O/^16^O ratio. The δ^13^C and δ^18^O values were normalised using a three-point calibration against international standards (IAEA-603 (δ^13^C = 2.5‰; δ^18^O = −2.4‰); IAEA–CO − 8 (δ^13^C = −5.8‰; δ^18^O = −22.7‰); and IAEA NBS 18 (δ^13^C == −5.014‰; δ^18^O = −23.2‰)). The in-house standard was USGS44 (δ^13^C = −42.2‰). Replicate analysis of USGS44 standards indicates that machine measurement error was c. ±0.1‰ for δ^13^C and ±0.2‰ for δ^18^O. Overall measurement precision was accessed through repeat analysis of an equid tooth enamel standard (±0.2‰ for δ^13^C and ±0.2‰ δ^18^O). The equid enamel standard (ID: MAXIMUS) was analysed alongside three of the seven batches analysed during the current study.

### Strontium isotope analysis of faunal tooth enamel and plant material

Archaeological enamel and modern plant samples were analysed for strontium (^87^Sr/^86^Sr) isotope analysis in the Department of Geosciences at the University of Cape Town, South Africa, following established procedures (Copeland et al. 2008)^137^. Prior to analysis, the plant samples were ashed in the Stable Isotope Laboratory at the Max Planck Institute of Geoanthropology, Jena, Germany, utilising the following protocol. Samples were left to dry and all dirt was removed via brushing by hand. Following this, plant material was cut into <1 cm pieces using sterile scissors, cleaned with isopropanol between the processing of each sample. Approximately 20 mg of plant material was then placed into silica crucibles and ashed in a muffle oven overnight at 800 °C.

Chemical sample preparation followed the protocol cited in Copeland et al.^137^ modified from Daniel and Pin^138^. Plant samples were dissolved in 4 ml of a 4:1 mixture of 48% 2B (two-bottle distilled; 28 M) HF: 65% 2B HNO_3_ and held at 140 °C for 48 h in closed Teflon beakers. Once dissolved, the samples were dried down and redissolved twice in 65% 2B HNO3 then subsequently redissolved once more in 1.5 ml of 2 M HNO_3_. Processing of the enamel samples prior to analysis followed the established procedure^139^. Powdered enamel samples were weighed into 7 ml Teflon beakers, 2–3 ml of 65% HNO_3_ was added, and the beakers were closed and kept at 140 °C for 1 h. Following complete sample dissolution, the samples were dried and taken up in 1.5 ml of 2 M HNO_3_. The strontium fraction was then isolated following the procedure in Pin et al.^139^ using Eichrom Sr. Spec resin, dried down, before being redissolved in 0.2% HNO^139^. Isolated Sr fractions were subsequently analysed as 200 ppb Sr solutions using the NuPlasma HR MC-ICP-MS. Repeat analysis of an in-house plant reference material Namiso 316 processed in parallel with the plant samples yielded a result of 0.720508 ± 0.000043 (*n* = 3) which is in good agreement with the average for the facility (0.720508 ± 0.000043; *n* = 21). The enamel samples were processed alongside repeats of the in-house carbonate reference material (NM95) and gave an average ^87^Sr/^86^Sr value of 0.7089015 ± 0.000028 (*n* = 24). This was in good agreement with the long-term average of this reference material at the facility of 0.708911 ± 0.000040 [2σ]; *n* = 414. All ^87^Sr/^86^Sr data presented are referenced to bracketing analyses of the international strontium isotope standard NIST SRM987 (^87^Sr/^86^Sr reference value of 0.710255). Sample values were corrected for instrumental mass fractionation using the exponential law and an ^86^Sr/^88^Sr of 0.1194 and isobaric ^87^Rb interferences were corrected using the measured ^85^Rb signal and the natural ^85^Rb/^87^Rb. Total procedural blanks processed with samples in this facility yielded background Sr levels <250 pg and were therefore negligible.

### Determining local bioavailable environmental strontium (^87^Sr/^86^Sr)

Our ^87^Sr/^86^Sr base map was modified from the global ^87^Sr/^86^Sr isoscape generated by Bataille et al.^100^. We also obtained modern plant samples of grass, shrub and tree leaves due to the strong relationship between ^87^Sr/^86^Sr values of plants and geological substrates^140^ from three locations from different geologies on the northern edge of the Thuringian Basin in order to confirm the accuracy of modelled ^87^Sr/^86^Sr values^100^ for the study region.

### Reporting summary

Further information on research design is available in the Nature Portfolio Reporting Summary linked to this article.

### Supplementary information

Supplementary Information

Description of Additional Supplementary Files

Supplementary Data 1

Supplementary Data 2

Supplementary Data 3

Reporting Summary

## Data Availability

All data generated or analysed during this study are included in the published article and its Supplementary Data files.
