# Supplementary material for: Multi-isotope reconstruction of Late Pleistocene large-herbivore biogeography and mobility patterns in Central Europe
Source: Commun Biol. 2024 May 14;7:568. doi: 10.1038/s42003-024-06233-2 (PMC11094090; doi:10.1038/s42003-024-06233-2)
Supplement: Supplementary file 1 — Supplementary Information [file 42003_2024_6233_MOESM1_ESM.pdf]

Supplementary Information for

Multi-isotope reconstruction of Late Pleistocene large-herbivore biogeography and mobility patterns in Central Europe

Phoebe Heddell-Stevens[1,2]*, Olaf Jöris[3,4], Kate Britton[5,6], Tim Matthies[3,4], Mary Lucas[1,7], Erin Scott[1], Petrus Le Roux[8], Harald Meller[9] and Patrick Roberts[1,10].

[1]Department of Archaeology, Max Planck Institute of Geoanthropology, Jena, Germany.
[2]Institute of Oriental Studies, Indo-European Studies, Prehistoric and Early Historical Archaeology, Friedrich Schiller University, Jena, Germany.
[3]Leibniz-Zentrum für Archäologie (LEIZA), MONREPOS Archaeological Research Centre and Museum for Human Behavioural Evolution, Neuwied, Germany.
[4]Institue of Ancient Studies, Johannes Gutenberg University Mainz, Mainz, Germany.
[5]Department of Archaeology, University of Aberdeen, Aberdeen, United Kingdom.
[6]Department of Human Evolution, Max Planck Institute for Evolutionary Anthropology, Leipzig, Germany.
[7]Arctic University Museum of Norway, Tromsø, Norway.
[8]Department of Geosciences, University of Cape Town, Cape Town, South Africa.
[9] State Office for Heritage Management and Archeology Saxony-Anhalt — State Museum of Prehistory, Halle, Germany.
[10]School of Social Sciences, University of Queensland, Brisbane, Australia.

*Corresponding author. Email: heddellstevens@gea.mpg.de

This PDF file includes:

Supplementary Note 1: Relevant faunal ecological and ethological information
Supplementary Note 2: Stable carbon, oxygen and strontium isotope analysis of herbivore tooth enamel in Central Europe – detailed background
Supplementary Note 3: Timing of herbivore tooth enamel formation and mineralisation
Supplementary Note 4: Site backgrounds and faunal assemblage information
Supplementary Tables 1 to 3
Supplementary References

Supplementary Note 1. Relevant faunal ecological and ethological information

Caribou and reindeer (*Rangifer tarandus*)

Diet and water intake. Extant *Rangifer tarandus* (including sub-species of caribou and reindeer) exhibit a large degree of plasticity in their feeding behaviour and are typically generalist feeders consuming a wide range of plants including tree browse, shrub leaves, bushes, grasses, mosses, mushrooms and lichen ([1]). Modern caribou tend to select for plants with high protein and fat content and high digestibility ([2–4]). Caribou exhibit diet shifts on a seasonal basis according to environmental parameters. During the colder months lichens are the major food source for many modern herds comprising 50-80% of winter diet with terrestrial varieties being the most commonly consumed (e.g. [5–8]). During the spring and summer months extant caribou may feed on young tree shoots and leaves, sedges and flowers as well as grasses, and in autumn mushrooms and berries are also consumed ([9–11]). *R. tarandus* are non-obligate drinkers, obtaining the majority of their water from through ingested leaf water.

Habitat. Habitat selection in extant *R. tarandus* populations is defined by predator avoidance, forage availability and climate conditions and snow cover ([3,12–15]). Among modern ungulates, they have the widest circumpolar distribution and are demonstrate the greatest cold tolerance ([3,5,16]). They are able to withstand relatively short summer and long, cold winters with temperatures of -50°C and snow cover several metres deep ([3,5,17]). Today, reindeer occupy a range of different environments that include sub-Arctic taiga, open plains, mountainous regions, boreal and deciduous forests, river and lake margins as well as polar deserts ([3,12,17,18]). For modern herds, shifts in habitat preference are typically seasonal ([19]). While variation in habitats exploited by exhibited different *Rangifer* sub-species in North America and Eurasia is large, some broad trends can be observed. Winter habitat correlates largely with snow depth and density and resulting access to forage, i.e. terrestrial lichens (e.g. [13,15,20]), while in the spring certain sub-species of *R. tarandus* (woodland and mountain caribou) move to open slopes to calve in order to protect against insects and predators ([13,21]). In autumn different populations have been observed seeking shelter in light forest at lower elevations ([3,22]).

Home range and mobility patterns. Caribou and reindeer populations today and in the recent past display huge variation in home range size and include both sedentary and migratory ecotypes (e.g.[3,5,15]). Home range as defined by Burt ([23p452]) is “that area traversed by the individual in its normal activities of food gathering, mating, and caring for young”. Home range size within populations can be variable and depends heavily on forage availability, distance between winter and summer ranges, predation and local herd size ([3,15,24,25]). While differences in home range size of hundreds of kilometres exist between and within sub-species of North American *R. tarandus* can be vast as the species incorporates both migratory and sedentary ecotypes, sometimes within the same herd ([26]). Seasonal migrations are defined as periodic, to-and-fro movements between two or more distinct seasonal ranges ([27]). Sedentary or resident behaviour is described as “comparatively short movements occurring within an area that is often frequented throughout an animal’s lifetime” ([26,28]). Predominantly sedentary ecotypes include woodland and mountain caribou. Home range sizes in mountain caribou typically vary between <100 and >800 km^2 ([15,29,30]), while in woodland caribou home range size can varying between 312 and

8,838 km^2 ([31]). Boreal caribou in Alberta, western Canada, are known to wander throughout the year, undertaking movements across ranges that average 710 km^2 ([30]). Central and Northern caribou ecotypes are partially migratory, with the later exhibiting variability in home range size, between 1,100 and 1,900 km^2 for some herds and 150 km^2 for others ([14,32]). Barren-ground caribou are largely migratory, and have home ranges that can cover several hundred thousand kilometres ([3,33,34]) and can average ~300 km one way during migrations ([26,35,36]). These movements are undertaken in order to exploit seasonally available resources, and forage availability as well as snow cover, climate conditions and seasonality, predator avoidance and topography all play a role in determining the direction, distance, timing and inter-annual continuity of migration ([3,27,37–39]). Migration routes can change from one year to the next, demonstrating the plasticity and potentially discontinuity in geographic ranges of extant herds ([40–44]). Barren-ground caribou undertake migrations in spring between April and May (prior to birth) and autumn, following the rut ([3,5,45,46]). In more mountainous regions, movements outside of migration may be altitudinal, and calving may take place in elevated areas ([13,15,47]). During the summer, modern caribou movements and range location are generally driven by foraging requirements and insect relief ([39,46]). During calving, caribou are typically the most dispersed and occupy the smallest ranges ([15,19,39,48]), while in winter animals are most widely distributed with the largest ranges ([39,49]).

Biology and social structure. While the social structure of modern *R. tarandus* herds also fluctuates according to the season, sexual segregation can be highly variable ([3,17,50]). The basic unit is the matriarchal winter herd which comprises of cows and juveniles of both sexes. These groups leave their winter range and migrate to their calving grounds during the spring ([33,46,51]). Females often to disperse in order to give birth to a calf in early summer, typically between May and early June, with births being highly synchronised ([3,15,17,43]). Matriarchal groups then move to their summer region where they are joined by the adult males ([17,27]). Reindeer then move to autumn breeding grounds, aggregating over the course of a few weeks from mid-September through October prior to rutting between October and November ([15,33,43,46]). Adult males may then leave the matriarchal herds and move to the winter range, and the autumn migration typically continues through November ([3,17,45,46]). Herds form twice a year, in spring and autumn and are often widely dispersed at other times of the year ([52,53]).

Wild horse (*Equus* sp.)

Diet and water intake. Extant wild horse populations, also referred to as free-ranging, free-roaming, or feral, are predominantly grazers, preferentially selecting graminoids but also consuming forbs, shrubs and browse to a lesser extent (e.g. [54,55]). Observed seasonal changes in diet are predominantly dictated by forage availability ([56,57]) and may include a higher consumption of woody plants during the winter months ([54,58,59]). However, in some modern feral herds, browse makes up the majority of the equid diet year-round ([60,61]). All equids are obligate drinkers, and as such must obtain the majority of their water through drinking ([62,63]). In the warm season they are dependent on open water sources the distribution of which strongly determines summer movements, spatial distribution and range size of equid herds ([64]). In the winter months, horses are able to hydrate by eating snow ([65,66]) and reducing the need to maintain close proximity to water sources, enabling them to range further in search of food ([64,67,68]).

Habitat. Modern-day free-ranging equids typically prefer flat, open areas such as grasslands ([64,69,70]) but can also be found in forest habitats ([64,71]). They are able to withstand cold, dry conditions, inhabiting steppe and desert regions ([72]), and can tolerate snow cover up to 60 cm depth for short periods, but are less tolerant of wetter climates ([56]). While in general, forage availability appears to be the best predictor of habitat use ([64,73–75]), terrain, slope, elevation (avoidance of high altitudes), distance to water as well as climate conditions also play key roles in habitat selection ([56,64,75,76]). Seasonal differences in habitat selection typically reflect availability of quality food sources and access to drinking water, snow cover and shelter from harsh winter winds, which often means sheltering within wooded areas ([64,71,77,78]). In spring and summer, lower mountain slopes, river basins and valley floors and open, flat areas are preferred, while in autumn and winter wild horses may preferentially select mid-slopes with north facing aspects mid-slopes in autumn and winter with a preference for north facing aspects ([79]).

Home range and mobility patterns. A wide variation in home range size has been reported within North American feral horse populations ([70] and references therein). Home range size in wild equids is largely dependent on resource availability ([70]). In mesic steppe grasslands home range size is typically smaller (e.g. 0.75-12 km^2) ([77]) than in arid regions, where it may vary from 290 to 1,357 km^2 ([80]). Meanwhile horses inhabiting forested areas have been reported to exhibit home ranges of 40.4 km^2 and 48.2 km^2 ([71,81]). Feral horses tend to exhibit high fidelity to home range location ([82]), with varying degrees of overlap observed between those of individual herds ([64,79]). Wild horses can be highly and movements across time and space appear predominantly to be driven by resource variation, and therefore are often seasonal ([64,70,75]). Winter ranges are often larger than summer ranges due to horses' ability to obtain drinking water from snow ([65,83]) and relative scarcity of resources during the winter months ([64,77,78,84]).

Biology and social structure. Wild equids inhabiting the same region will form herds that share a home range and follow similar movement patterns. Within herds are bachelor and harem groups, which include natal bands. Bachelor groups are all-stallion groups formed of surplus males while harem groups are relatively stable groups of one to multiple females and young born that year and one or two tenured stallions ([84–86]). Groups are most widely dispersed during winter months when forage abundance is lowest. In the spring, bands congregate and females give birth to young, followed by the rut ([87]). In the late spring and summer equids break into harems and bachelor bands, becoming more dispersed as food supply decreases ([88]).

Elephant (*Loxodonta africana, Loxodonta cyclotis, Elephas maximus)*

Diet and water intake. Mammoth ecology and behaviour are largely inferred from data pertaining to historical and modern elephant populations (the African savannah or bush elephant, African forest elephant and Asian elephant), although studies based on long-term observations are limited (but see [89]). Extant elephants are typically generalist feeders, consuming ~150 kg of forage daily ([90,91]). Differences in diet breadth have been identified between species, with some populations consume more leaves, bark and fruits than others, in particular those inhabiting tropical forests exhibit a broader diet ([92–95]). Across all species dietary shifts appear to take place

largely in response changing forage availability, i.e. seasonal availability of preferred plant species ([96–100]). African forest elephants and Asian elephants consume woody plant material, browse and grasses throughout the year, with consumption of the latter increasing during the wet season ([101,102]). Like extant proboscideans, mammoth would have been obligate drinkers, with an estimated required intake of 100-300 l of water a day, based requirements of modern elephants ([103,104]). This means a reliance on access to water sources and on plants with high sodium content ([90]).

Habitat. Modern elephant populations occupy a variety of habitats from grassland to desert environments as well as tropical forests (e.g. [105–108]). While African forest elephants and Asian elephants predominantly occupy woodland and forest habitats, the African savannah elephant is also adapted to closed forest as well open environments ([100,101,108–110]). Across the different species habitat selection is largely influenced by forage distribution, water availability and amount of shade (e.g. [99,111–114]). Owing to these needs, elephants will often select riverine or waterside environments, particularly during the dry season ([94,100,108,110,115]). In contrast, during the wet season, cow herds of African elephants select more open *Acacia* habitats ([108]).

Home range and mobility patterns. Home range size and distance of movements in extant proboscideans is primarily correlated with availability of water sources, food supply, seasonal changes as well as the availability of suitable habitats ([101,105,108,116]). Extant elephants are typically highly mobile, due to their daily requirement for large quantities of forage and water. Home range size in modern African savannah elephant can vary between populations from 15 to 3,700 km^2 ([107]), with individuals inhabiting open areas occupying larger home ranges in general, typically between 90-800 km^2 ([94,117,118]). Estimated home range size of the Asian elephant is ~100 to 300 km^2 ([105]) although more research is need. Certain populations may undertake long-distance seasonal migrations ([94,119,120]), however, these are likely significantly reduced today in comparison to the past due to increasing habitat destruction ([107]). Intra-herd differences in mobility patterns of African savannah elephants show that not all individuals in a region will undertake migrations ([121,122]). Some individuals migrate opportunistically and not every year, while others migrate between distinct seasonal ranges correlating with wet and dry seasons ([121,122]). Furthermore, elephants exhibit flexibility in mobility patterns that can shift between generations ([108,115,116]). There may also be sex-related differences in seasonal movements, i.e. female African savannah elephants often occupy predictable ranges during the dry season while migrating long distances during the wet season ([119,120]).

Social organisation and biology. The basic social unit for elephants is the mother-offspring unit ([94,101,107,123,124]) and both African elephants ([94,123]) and Asian elephants ([101,124]) have been identified as forming matriarchal ‘kinship groups’ comprising of two of three of these units. While the is no pronounced breeding season in elephants, oestrus is reflective of resource availability and rainfall therefore single calf births can occur throughout the year ([90,94,109,125]). Once males reach puberty at ~9-18 years of age they separate over a number of years from these familial herds and disperse locally ([89,123,124]). After dispersing, adult males are solitary, occasionally associating with other males and matriarchal groups to mate ([123–126]).

Supplementary Note 2: Stable carbon, oxygen and strontium isotope analysis of herbivore tooth enamel in Central Europe for mobility studies - detailed background

Strontium ($^{87}Sr/^{86}Sr$) isotope analysis of herbivore tooth enamel in Central Europe

The majority of bioavailable (water-soluble) strontium ($^{87}Sr/^{86}Sr$) isotope ratios in the environment derive primarily from underlaying bedrock ([127]). The stable but radiogenic isotope ^{87}Sr is a product of the radioactive decay of rubidium (^{87}Rb), while ^{86}Sr is stable. The final ratio of ^{87}Sr to ^{86}Sr in bedrock is dictated by the age of the rock (older rocks exhibit higher $^{87}Sr/^{86}Sr$ ratios), rate of weathering, chemical composition and mineral component ([127–129]). As strontium weathers it is incorporated into overlaying sediments and ground waters, and in turn, is taken up by local plants which are ingested by herbivores ([130–132]). Strontium substitutes for calcium in the mineral component of tooth enamel, hydroxyapatite ([133]), and as such, $^{87}Sr/^{86}Sr$ ratios in herbivore enamel reflect those of the area in which the individual sourced its forage. Unlike other isotope systems, there is very little fractionation of $^{87}Sr/^{86}Sr$ as it moves through the biosphere, enhancing its suitability as a mobility proxy (e.g. [134–136]).

When bioavailable strontium isotope ratios for a region are known, comparisons between the $^{87}Sr/^{86}Sr$ of individuals and those of the modelled $^{87}Sr/^{86}Sr$ baseline can enable the use of strontium as a mobility proxy (for overviews see [128,129,137]). Recent developments in statistical modelling have produced both global- and regional-scale maps or 'isoscapes' of environmental bioavailable $^{87}Sr/^{86}Sr$ ([138–142]) that enable researchers to move beyond the identification of local and non-local individuals towards mapping animal movements onto the landscape, through visual comparisons and spatial assignment programs (e.g. [143,144]). Due to the incremental nature of tooth enamel mineralization, movements made by an individual across different lithologies during the period of mineralisation are recorded in time-resolved sequence within the tooth (Supplementary Note 3). As a result, intra-tooth sampling of enamel provides information pertaining to an individual's movement on a seasonal scale, and the applicability of this approach has been demonstrated in a number of studies from Late Pleistocene European contexts (e.g. [143–145]).

However, this approach is constrained by a number of factors. It relies on there being sufficient differences in bioavailable $^{87}Sr/^{86}Sr$ ratios between the different places of residence, to enable movements to be detected ([137]). Therefore, homogenous lithologies over large areas which can obscure individual movement over long distance. Conversely, highly heterogenous lithologies in a locality may produce an averaged $^{87}Sr/^{86}Sr$ signal in enamel as individuals move across different geologies within shorter time frames (i.e. faster than the rate of enamel mineralization) ([137]). Furthermore, the relationship between $^{87}Sr/^{86}Sr$ ratios of local geology and overlaying soils and plants is not always straightforward ([129,146,147]). Single rocks such as granite can exhibit large ranges of $^{87}Sr/^{86}Sr$ ratios as a result of different minerals within the rock, thereby producing a wide range of 'local' bioavailable strontium values ([129]). Aeolian deposits such as loess as well as cover sands and glacial moraines can travel hundreds of kilometres resulting in differences between bioavailable $^{87}Sr/^{86}Sr$ ratios and those of the underlaying geology ([129,147]).

To address this, analysis of spatially-constrained materials including modern soil, water, plants, invertebrates and small mammals, as well as archaeological material, may be undertaken to establish local bioavailable $^{87}Sr/^{86}Sr$ ratios (for discussions see [137,146–148]). There is ongoing discussion regarding which type of samples best represents local bioavailable $^{87}Sr/^{86}Sr$ and questions remain as to the applicability of modern sampling to establish ancient strontium baselines ([129,130,148]) with more work needed to refine these approaches.

Stable oxygen ($\delta^{18}O$) isotope analysis of herbivore tooth enamel in Central Europe

At northern hemisphere mid-latitudes, environmental $\delta^{18}O$ broadly reflects $\delta^{18}O$ of precipitation, which is primarily influenced by local air temperature and rain-out effect ([149–151]). In this region, $\delta^{18}O$ values of local precipitation display seasonal variation, i.e. lower $\delta^{18}O$ values during the colder months and higher $\delta^{18}O$ values during the warmer months ([149,152,153]). While smaller rivers and lakes tend to be locally-fed by meteoric water and therefore more faithfully reflect $\delta^{18}O$ of local precipitation ([154–156]), there are a number of factors that can affect this relationship. These include the influence of evaporation, transpiration and outflow rates in open versus closed lakes ([157]), runoff from elevated areas including snowmelt ([158–160]), and input from groundwater ([161]). Groundwater and snow are typically depleted in ^{18}O compared to local precipitation ([154,155,160,162]), as a result, the incorporation of these waters into rivers and streams can result in lower summer $\delta^{18}O$ values ([154,163,164]). Stable oxygen isotope values of plant leaf water are enriched compared to those of local precipitation due to evapotranspiration processes ([165]). Leaf water $\delta^{18}O$ values are sensitive to aridity, with increasing aridity resulting in further enrichment of leaf water $\delta^{18}O$ values, and are therefore reflective of seasonal climate shifts at temperate mid-latitudes ([165–167]).

Stable oxygen isotope values of herbivore enamel apatite are controlled by the isotopic composition of body water which predominantly reflects that of total ingested water ([154,168–170]). Obligate drinkers, including horses and most likely mammoth, obtain the majority of their body water via drinking water, and as such, their enamel $\delta^{18}O$ values reflect those of consumed water sources and therefore more likely to reflect $\delta^{18}O$ values of local precipitation ([168,170]). In non-obligate drinkers, including cervids, $\delta^{18}O$ values of tooth enamel largely reflect evaporatively ^{18}O-enriched ingested leaf water ([171,172]) which have also been shown to track relative seasonal shifts in $\delta^{18}O$ of meteoric water ([173]). Therefore, medium- and large-herbivore obligate and non-obligate drinkers have the potential to qualitatively track seasonal changes in $\delta^{18}O$ of meteoric water ([174–176]). Certain species, including horses, that can consume ^{18}O-dpeleted snow to obtain drinking water ([65,83]) are still be expected to exhibit seasonal variation in $\delta^{18}O$ values of incrementally forming tissues. Intra-tooth $\delta^{18}O$ values can therefore be employed as a seasonal indicator and when combined with other isotope analyses, they enable the detection of seasonal-scale movements ($^{87}Sr/^{86}Sr$) and dietary shifts ($\delta^{13}C$) (e.g.[136,177]). Animals that exhibit a semi-sinusoidal pattern in their intra-tooth $\delta^{18}O$ measurements are interpreted as having experienced local-scale seasonal variations in climatic parameters and therefore as being relatively sedentary ([134]). In contrast, a relatively attenuated seasonal intra-tooth enamel $\delta^{18}O$ can be indicative of rapid, seasonal long-distance migrations by an individual ([134,178–180]). As a result, $\delta^{18}O$ values of herbivore enamel can be employed as an additional indicator of individual mobility in past populations, anchoring $^{87}Sr/^{86}Sr$ data in a seasonal context (e.g. [136,175,181,182]).

Stable carbon ($\delta^{13}C$) isotope analysis of herbivore tooth enamel in Central Europe

During the late Pleistocene, Western and Central Europe was dominated by plants utilising the C_3 photosynthetic pathway. C_3 plants include temperate species such as shade-loving grasses, herbs, woody shrubs and most tree taxa. In terrestrial environments these plants typically exhibit $\delta^{13}C$ values between approximately -24 and -36‰ ([183]). The major environmental factors influencing $\delta^{13}C$ values of C_3 plants are aridity, water stress, temperature, light and nutrient availability, salinity and altitude ([184–187]). These factors result in discrimination against ^{13}C which produces differences in $\delta^{13}C$ values between different types of plants ([184,187,188]). Within an ecosystem, there is a trend towards more negative $\delta^{13}C$ values (typically <-28‰) in plants within closed forest environments, known as the 'canopy effect', which is the $\delta^{13}C$-depletion of CO_2 relative to that of the atmosphere as a result of the CO_2 generated by the respiration and decomposition of organic matter, low light intensity and carbon recycling ([183,189,190]). In contrast, plants living in open grassland and steppe-tundra environments display relatively enriched $\delta^{13}C$ values >28‰ ([183]). Notably, lichens, a major food source of modern reindeer, are typically enriched in ^{13}C relative to other vascular C_3 plants (trees and grasses) in the same biome ([191,192]).

Herbivore enamel bioapatite $\delta^{13}C$ values record the dietary proportion of different C_3 plants ([193–195]). As herbivores take up stable carbon isotopes via ingested forage these variations in plant $\delta^{13}C$ can then be used to identify the dietary contribution of different plants to animal diets (e.g. [135,183,196–197]). Physiological traits, for example, ruminant versus non-ruminant species, also play a role in determining herbivore enamel $\delta^{13}C$ values however overall, these factors are recognised to have a much smaller effect on the final $\delta^{13}C$ values in comparison to diet ([183,198,199]). As the end values of C_3 plants in different environments are not absolute, interpretations of herbivore enamel stable carbon isotope values are context specific. In herbivores, enamel carbonate $\delta^{13}C$ values are fractionated around +14.6‰ relative to dietary plants therefore result fossil taxa feeding exclusively on C_3 plants will exhibit $\delta^{13}C$ values between ~20‰ to ~6‰ ([193,200]). In northern hemisphere temperate mid-latitudes C_3-plant dominated environments during the late Pleistocene, the incorporation of large amounts of lichen into herbivore diets is indicated by high $\delta^{13}C$ values (11‰ and above) relative to those of grazers (ca. -14‰ to 10‰), which are elevated compared to those of browsers (ca. <-11‰) ([183,191,192]). Feeding in dense forest habitat is expected to produce $\delta^{13}C$ values of -14‰ and below ([183,201,202]). Analysis of intra-tooth stable carbon isotope ratios alongside $\delta^{18}O$ values enables researchers to identify seasonal dietary shifts which in turn can provide support for seasonal movements indicated by $^{87}Sr/^{86}Sr$ data (e.g. [136,182,203]).

Supplementary Note 3. Timing of herbivore tooth enamel formation

In hypsodont herbivores tooth enamel forms incrementally from the cusp to the apex (the root) over a period of months or years ([204]). The timing of mineralisation depends on the tooth and species in question. It is during this process that time-resolved isotopic data pertaining to dietary habits and mobility patterns of an individual during this period is captured in the tooth enamel bioapatite. Once

mineralised, enamel bioapatite compositions are not replaced during an individual's lifetime its dense crystalline structure means it is relatively resistant to diagenetic alteration compared to other skeletal tissues ([205–207]). There are systematic differences in the timing of enamel mineralisation between teeth in a single jaw and also between species (e.g. [204]). In reindeer and horses, teeth in the jaw form roughly in sequence (with varying degrees of overlap) therefore sampling multiple teeth from one individual provides a longer continuous record of isotopic data (Supplementary Table 3) ([208]).

The exact timing of enamel mineralisation is not currently known for reindeer and is therefore estimated on the basis of comparisons with other cervids (Supplementary Table 1) ([209,210]). Predicted timing of enamel mineralisation in reindeer teeth is further supported by intra-tooth isotope studies on modern caribou populations ([134,211]). For this study we targeted the later forming permanent second (M2) and third molar (M3) in our study to avoid any influence of an isotopic weaning signal from mother's milk as modern caribou calves are generally weaned around two years of age ([212,213]). In *Rangifer* the M2 is expected to form between <3.5 and 9 months, and the M3 between 9 and <18 months ([209,210]).

Supplementary Table 1: Tooth enamel mineralization times (in months) of *Rangifer tarandus*, *Equus* sp. and *Mammuthus primigenius* permanent molar teeth sampled in this study. *Rangifer* mineralisation times are adapted from Brown and Chapman ([209]), Britton et al. ([134]) and Price et al. ([178]). Timing of mineralisation for *Equus* sp. is adapted from Hoppe et al. ([208]). Estimated timing of *M. primigenius* enamel mineralisation is estimated from modern elephant data ([214,215]).

	**M1**		**M2**		**M3**		**M4**	**M4**
	Onset	*Completion*	*Onset*	*Completion*	*Onset*	*Completion*	*Onset*	*Completion*
R. tarandus			<3	<9	~9	~18		
Equus sp.	0.5 (±1)	23 (±3)	7 (±1.5)	37 (±3)	21 (±3)	55 (±2)		
M. primigenius							~3 (years)	~15 (years)

The timing of enamel mineralisation in modern equids has been directly assessed (Supplementary Table 1) (e.g. [208,216,217]). We selected preferentially selected M2 and M3 teeth, again, in order to avoid a capturing a weaning signal as weaning typically occurs prior to M2 enamel mineralisation, however in one case it was necessary to sample a permanent first molar (M1) from Breitenbach as no further M3s were available. In M1 teeth, enamel mineralisation in horses begins at 0.5 (±1) months ending at 23 (±3) months and comprising of a total of ~2 years ([208,218]). Mineralisation in the M2 occurs between 7 (±1.5) and 37 (±3) months (~35 months in total), while in M3 teeth mineralisation takes place between 21 (±3) and 55 (±2) months (a total of ~34 months) ([208,218]).

Information on enamel formation and mineralisation times for *Mammuthus primigenius* is primarily based on data from extant African elephants (*Loxodonta Africana*) (Supplementary Table 1) ([214]). Mammoth teeth are comprised of multiple parallel enamel plates, or lamella, which most likely formed incrementally from crown to root ([219]). We sampled an M1 tooth as the most intake specimen available in the

Königsaue assemblage. Utilising Law's calibrations ([214,215]), Metcalfe et al. ([215]) estimates that M1 crown formation in mammoths began at ~3 years and was completed at ~15 years, comprising of a ~12-year period. Metcalfe and Longstaffe ([220]) use histological and isotopic data to estimate the enamel extension rates (growth in the height of the tooth) to average ~13-14 mm per year in the Columbian mammoth, however this may vary between individuals by between ~0.5 and 2.3 cm per year ([220–224]). We assume a similar timing of lengthwise enamel formation for the Königsaue individual. Taking into account wear and loss of enamel during burial and subsequent handling, the height of the tooth crown (80 mm) would then represent a period of enamel mineralisation of ~5.5 years.

For all species and teeth, enamel wear begins when the tooth eruptions and continues throughout the individuals' lifetime. While the amount of wear differs depending on diet and age of an individual, all teeth, and therefore the isotopic signal, will be reduced as a result. The timing of enamel mineralisation for full, unworn teeth sampled for this study is given in Supplementary Table 1. These include permanent first, second and third molars of horses, second and third permanent molars of reindeer and the first adult molar in mammoth.

Supplementary Note 4: Site background and faunal assemblage information

Königsaue

Site background. Königsaue (51°49'N, 11°24'E) is a Middle Palaeolithic open-air site to the north of the town of Aschersleben in Saxony-Anhalt, eastern Germany, and approximately 15 km northeast of the Harz Mountains. The site was located on the shore of the Late Pleistocene to Holocene Aschersleben Lake, roughly 12 km in length, and was exposed in the 1970s during lignite open-cast mining ([225]). Once mining ceased in 1996 the pit was flooded and became the present-day Königsauer See. During mining, rescue excavations were carried out at the site by Mania and Toepfer between 1963 and 1964 ([225]), during which three separate archaeological layers have been identified, Layers A, B and C (oldest to youngest). All these layers are found within the lake shore deposits of sediment Cycle Ib.

This study is concerned with the faunal material from Layer A (KÖA) which is the richest of the three archaeological layers regarding the amount of faunal remains as well as of Middle Palaeolithic lithic artefacts that place the assemblage into a context of the Late Middle Palaeolithic *Keilmessergruppen* (KMG) ([226] and references therein). In addition to the presence of stone tools, hominin presence in KÖA is attested to by the identification of pieces of birch-bark pitch, one of which features a hominin thumb print and the imprint of a wooden haft ([225,227]). The lithic assemblage of KÖA comprises 1,478 flint and 12 quartzite artefacts ([225]).

Stratigraphy and Dating. With totally up to more than 20 m in thickness Königsaue comprises one of the most complete sequences of the Late Pleistocene in the Northern European Plains - preserved in a depression which has been carved during a Middle Pleistocene ice advance ([225,226,228–231]). Interglacial deposits at the base (Cycle Ia) are assigned to the Last Interglacial, i.e. the Eemian. They are followed by sequence of Early Weichselian, i.e. later MIS 5 interstadial deposits (Cycles Ia_2, Ib,

IIa, and IIb), an interpleniglacial (i.e. MIS 3) sequence (Cycles III, IVa, IVb, V) and Late Glacial (Cycles VII and VIII) to Holocene (Cycle IX) deposits. The two Last Glacial pleniglacial periods of MIS 4 and 2 are represented by Cycles IIb and VI respectively, characterized by severe pleniglacial conditions. Based on this stratigraphy the archaeological horizons are assigned to the second Early Weichselian interstadial, corresponding to the palynologically defined Odderade interstadial (MIS 5a). Their age is thus to be estimated of ca. 80 kyr ([225,226,228–231]). This estimate is supported by a series of infinite radiocarbon dates > 45.0 (B-626) – 55.8 (GrN-5698) uncalibrated (uncal) BP from Cycle Ib materials and a radiocarbon date of 60.1 +1.4/-1.2 uncal BP (GrN-7001) from the same horizon as well as by a radiocarbon date of 49.2 +4.1/-2.7 uncal BP (GrN-7078) from the much younger, MIS 3 Cycle III deposits ([232]). These dates conflict, however, with further ^{14}C-dating attempts on bone ([231]) that were undertaken on two pieces of birch-bark pitch from cultural contexts KÖA and KÖB, with the Layer A sample returned a date of 43.8 ± 2.1 uncal BP (64:1/0, $\delta^{13}C$ = -26.5‰) (OxA-7I24) ([233]), arguably too young when referred to the stratigraphic sequence. (cf. discussions in [231,234–237]). A more recent date was obtained from a reindeer femur from level A dating to 41.82 ± 390 uncal BP (MAMS-24487) using ultrafiltration pre-treatment ([231]).

Faunal assemblage. Faunal analysis of the KÖA assemblage was undertaken by Mania and Toepfer ([225]). The faunal assemblage from Layer A comprises of fairly even numbers of medium-large and mega herbivores, including reindeer (*Rangifer tarandus*) (MNI=5), horse (*Equus* sp.) (MNI=4) and mammoth (*Mammuthus primigenius*) (MNI=4), as well as bison (*Bison priscus*) (MNI=3), and a single carnivore species, cave hyena (*Crocuta spelaea*) (MNI=2) (Supplementary Table 2) ([225]). The lake sediments in which the fossil remains were located comprised of humus-rich sandy peat layers which were not conducive to the preservation of faunal material, leaving many of them completely decalcified. However, certain sediments in horizon Ib enabled greater preservation of bones and teeth, particularly the Bruchwald peat layers, from which the faunal material for this study was selected ([225]). The faunal remains from KÖA are dispersed over a large area along the former lake shore, and comprises of isolated skeletal elements with a lack of cutmarked material ([225]). As such it is unclear to what extent the material accumulated due human activity or anthropogenic processes. Furthermore, the effects of weathering are present on a large number of the fossil finds, further contributing to survival rates of more fragile elements ([225]). According to the excavators, the accumulation of faunal remains by hominins at the site is inferred on the basis of the spatial relationship between the position of the lithic artefacts and the faunal material ([225]). Season-of-death was established previously for three reindeer individuals from KÖA on the basis of tooth wear stages of three permanent third molars ([238]), which indicated that these individuals were approximately 24, 25 and 36 months of age when killed ([225]). If these animals were born in late spring-early summer (June-July) this would mean they died between June and August.

Supplementary Table 2: Qualitative and quantitative composition and sample information for the Königsaue Layer A faunal assemblage.
Faunal counts including number of identified specimen (NISP and minimum number of individuals (MNI) were undertaken by Topfer ([225,231]). The number of individuals sampled, sample material (specific teeth) and individual sample numbers for the current study are

provided. The museum accession number (given for sampled elements) is the catalogue number designated by the State Museum of Prehistory, Halle, Germany.

**Species**	**NISP**	**MNI**	**Number of individuals sampled**	**Sample material**	**Individual sample number**	**Museum accession number**
Mammuthus primigenius	6	4	1	M4	KÖA_09	Kö A 27.2
Coelodonta antiquitatis	3	1				
Dicerorhinus hemitoechus	1	1				
Bison priscus	74	3				
Equus sp.	46	4	2	M2-M3	KÖA_01/02	Kö A 15.5
				M2-M3	KÖA_07/08	NA
Equus (Asinus) hydruntinus	3	1				
Cervus elaphus	2	1				
Rangifer tarandus	71	5	3	M3	KÖA_04	Kö. A. 9.1.64
				M3	KÖA_05	Kö. A.
				M3	KÖA_06	Kö. A. T
Crocuta spelaea	6	2				
**Total**	**212**	**22**	**6**			

Breitenbach

Site background. Breitenbach (Schneidemühle) (51°00'31.25"N, 12°05'06.75"E) is an Early Upper Palaeolithic open-air site located to the northeast of the village of Breitenbach, Sachsen-Anhalt, on the banks of the Aga riverlet, a right tributary to the Weisse Elster river. The site was discovered in 1924 during construction work and preliminary excavations were undertaken in 1925 by Niklasson of the Landesanstalt für Vorgeschichte, Halle/Saale, before excavations began later the same month led by Götze (Staatlisches Berliner Museum für Völkerkunde) ([239]). Large-scale excavations were conducted by Niklasson in 1927, uncovering an area of ~400 m^2 ([239,240]). This material – labelled Breitenbach A – is stored in the Landesamt für Denkmalpflege und Archäologie (LDA) Sachsen-Anhalt in Halle/Saale, whereas the material of a private collection, Breitenbach B, most likely deriving from the nearby Niklasson W trench limits, was sold in the 1950s to the German National Museum in Nürnberg. Following small-scale sondages in GDR time ([240]) and in 2004 and 2005 ([241]), ongoing large-scale investigations at the site restarted in 2009 in cooperation of the MONREPOS Archaeological Research Centre and Museum for Human Behavioural Evolution, a department of the Leibniz-Zentrum für Archäologie (LEIZA) and the LDA Sachsen-Anhalt., estimating the site's total spatial extension to around 10,000 m^2 ([242]). The lithic assemblage and radiometric dating assign the material to the late Aurignacian ([243,244]). A small number of tools made from organic material (bone and antler) were also discovered, and notable finds include a number of perforated Arctic fox canines ([242–246]). The site was initially interpreted as a base camp which was occupied for multiple and/or longer periods of time ([242,245,247]).

Stratigraphy and Dating. The Breitenbach assemblages include numerous lithic artefacts of Aurignacian type and evidence of intensive use of fire indicate "intense

human settlement activity" ([242–244,246,248]). The Aurignacian sequence is buried below a 2-3 m thick sterile loess sequence attributed to the last pleniglacial ([243]). A series of initial radiocarbon measurements of animal bone from both assemblages, Breitenbach A and Breitenbach B, produced age estimates too young for the cultural contexts, indicative of unsolved sample contamination problems in the open-air farming environment. The most recent dating efforts confirmed the dating problems due to overall low collagen yields, but in one case, a reindeer astragalus from the Breitenbach A collection, produced a radiocarbon date of 29.65 ± 280 uncal BP (OxA-21087) ([247]). This age estimate is in agreement with age estimates available for the Aurignacian to Mid-Upper Palaeolithic transition in Central Europe ([249]) and is slightly older than the oldest age estimates of Breitenbach material presented earlier ([240,250]).

Breitenbach A faunal assemblage. There is limited published information regarding the faunal material from Breitenbach. The Breitenbach A faunal assemblage includes the finds from Niklasson's excavations as well as a number of surface finds ([239,251]). The Breitenbach B faunal assemblage includes material obtained during unauthorized excavations at the site during the 1920 ([252]) and as such was not sampled as part of this study. The Breitenbach A faunal material is currently being studied as part of an ongoing PhD by Matthies ([253]). The current evaluation of the Breitenbach A material indicates that part of the assemblage, including the majority of the mammoth material, likely originated from late Middle Pleistocene deposits, some 20-30 cm below the archaeological layers ([247]), and appears to have accumulated naturally. However, the different status of surface preservation of some of the mammoth remains, suggests that mammoth were also present to some extent in the local environment during the period of human occupation, although it remains unclear whether mammoth hunting took place at the site ([253]). A lack of collagen preservation prevents radiocarbon dating hampering further attempts at age determinations. The recent MONREPOS field campaigns (2009 – ongoing) have uncovered further faunal material dominated by reindeer, Arctic hare and Arctic fox remains; with deviations in NISP and MNI counts (Supplementary Table 3) ([253]). The higher counts of Arctic hare and Arctic fox remains during recent field campaigns may – to some degree – be explained by refined modern-day excavation standards, whereas the Breitenbach A and B assemblages are biased by an overrepresentation of larger finds compared to the near absence of small objects, including fauna. While zooarchaeological analysis of this material is ongoing, taphonomic and seasonality data is currently limited. Evidence for anthropogenic modification has been identified on canid and reindeer material in the form of perforated canines and butchery patterns related to marrow extraction respectively ([254]). Preservation is generally poor and season-of-death information can so far only be determined for one *R. tarandus* individual on the basis of an unfused proximal phalanx ([254]). According to Hufthammer ([255]) and Pasda ([256]), fusing of the proximal epiphysis does not begin prior to the 6th or 7th month of life, which points to a late autumn or winter season of death.

Supplementary Table 3: Qualitative and quantitative composition and sample information for the Breitenbach A faunal assemblage.
Faunal counts including number of identified specimen (NISP and minimum number of individuals (MNI) were undertaken by Matthies ([254]). The number of individuals sampled, sample material (specific teeth) and individual sample numbers for the current study are

provided. Find locations refer to Niklasson's ([239]) excavation grid of 1/2 metre quadrants. The museum accession numbers are the same as the find locations.

**Species**	**NISP**	**MNI**	**Number of individuals sampled**	**Sample material**	**Individual**	**Find locations (quadrant)/ museum accession number**
Mammuthus primigenius	438	3				
Coelodonta antiquitatis	7	1				
Cervus elaphus	2	1				
Equus sp.	70	2	3	M1	BRE_01	K I
				M3	BRE_10	H 7 m
				M3	BRE_11	150-200 J
Rangifer tarandus	400	12	7	M2-M3	BRE_02/03	G v.3/G v.4
				M3	BRE_04	L 1
				M3	BRE_05	*No label*
				M3	BRE_06	D 5m
				M3	BRE_07	D 9m
				M3	BRE_08	B 4m
				M3	BRE_09	Y VIII (4)
Panthera spelea	4	1				
Crocuta spelea	2	1				
Canis lupus	60	3				
Vulpes alopex	155	10				
Lepus timidus	73	7				
**Total**	**1211**	**41**	**10**			

Supplementary References

1. Webber, Q. M. R., Ferraro, K. M., Hendrix, J. G. & Vander Wal, E. What do caribou eat? A review of the literature on caribou diet. *Can J Zool* **100**, 197–207 (2022).
2. White, G. Foraging patterns and their multiplier effects on productivity of northern ungulates. *Oikos* **40**, 377–84 (1983).
3. Geist, V. *Deer of the World: Their Evolution, Behaviour, and Ecology*. (Swan Hill Press, Shrewsbury, 1999).
4. Spiess, A. E. *Reindeer and Cairbou Hunters: An Archaeological Study*. (Academic Press, New York, 1979).
5. Kelsall, J. P. *The Migratory Barren-Ground Caribou of Canada*. (Queen's Printer, Otawa, 1968).
6. Rettie, W. J., Sheard, J. W. & Messier, F. Identification and description of forested vegetation communities available to woodland caribou: relating wildlife habitat to forest cover data. *For Ecol Manage* **93**, 245–260 (1997).
7. Danell, K., Mikael Utsi, P., Thomas Palo, R. & Eriksson, O. Food plant selection by reindeer during winter in relation to plant quality. *Ecography* **17**, 153–158 (1994).
8. Joly, K. & Cameron, M. D. Early fall and late winter diets of migratory caribou in northwest Alaska. *Rangifer* **38**, 27–38 (2018).
9. Nieminen, M. & Heiskari, U. Diets of freely grazing and captive reindeer during summer and winter. *Rangifer* **9**, 17 (1989).
10. Thompson, I. D. *et al.* Factors influencing the seasonal diet selection by woodland caribou (*Rangifer tarandus tarandus*) in boreal forests in Ontario. *Can J Zool* **93**, 87–98 (2015).
11. Skogland, T. Wild reindeer foraging-niche organization. *Holarct Ecol* **7**, 345–379 (1984).
12. White, R. G. & Trudell, J. Habitat preferences and forage consumption by reindeer and caribou near Atkasook, Alaska. *Arctic and Alpine Research* **12**, 511–529 (1980).
13. Nobert, B. R., Milligan, S., Stenhouse, G. B. & Finnegan, L. Seeking sanctuary: the neonatal calving period among central mountain woodland caribou (*Rangifer tarandus caribou*). *Can J Zool* **94**, 837–851 (2016).
14. Poole, K. G., Heard, D. C. & Mowat, G. Habitat use by woodland caribou near Takla Lake in central British Columbia. *Can J Zool* **78**, 1552–1561 (2000).
15. Cichowski, D., Kinley, T. & Churchill, B. Caribou, *Rangifer Tarandus.* in *Accounts and Measures for Managing Identified Wildlife*: Accounts v2004 (ed. Paige, K.) 224–252 (Ministry of Water, Land and Air Protection, British Columbia, 2004
16. Russell, H. J. *The Nature of Caribou: Spirit of the North*. (Greystone Books, Vancouver, 1998).
17. Fontana, L. *Reindeer Hunters of the Ice Age in Europe*. (Springer International Publishing, Cham, 2022).
18. Banfield, A. W. F. A revision of the reindeer and caribou genus Rangifer. *Bull-Natl Mus Can* **117**, (1961).
19. Ferguson, S. H. & Elkie, P. C. Seasonal movement patterns of woodland caribou (*Rangifer tarandus caribou*). *J Zool* **262**, 125–134 (2004).

20. Bjørkvoll, E., Pedersen, B., Hytteborn. I. S. & Langvat, R. Seasonal and Interannual Dietary Variation during Winter in Female Svalbard Reindeer (*Rangifer tarandus platyrhynchus*). *Arct Antarct Alp Res* **41**, 88–96 (2009).
21. Bergerud, A. T., Butler, H. E. & Miller, D. R. Antipredator tactics of calving caribou: dispersion in mountains. *Can J Zool* **62**, 1566–1575 (1984).
22. Nobert, B. R., Milligan, S., Stenhouse, G. B. & Finnegan, L. Seeking sanctuary: the neonatal calving period among central mountain woodland caribou (*Rangifer tarandus caribou*). *Can J Zool* **94**, 837–851 (2016).
23. Burt, W. H. Territoriality and Home Range Concepts as Applied to Mammals. *J Mammal* **24**, 346 (1943).
24. Gunn, A. & Miller, F. L. Traditional behaviour and fidelity to caribou calving grounds by barren-ground caribou. *Rangifer* **6**, 151 (1986).
25. Hemming, J. E. *The Distribution and Movement Patterns of Caribou in Alaska*. (Published by Alaska Department of Fish and Game, Alaska, 1971).
26. Theoret, J. *et al.* Seasonal movements in caribou ecotypes of Western Canada. *Mov Ecol* **10**, 12 (2022).
27. Joly, K. *et al.* Caribou and reindeer migrations in the changing Arctic. *Animal Migration* **8**, 156–167 (2021).
28. Roshier, D. & Reid, J. On animal distributions in dynamic landscapes. *Ecography* **26**, 539–544 (2003).
29. Stevenson, S. K. *et al. Mountain Caribou in Managed Forests: Recommendations for Managers*. (Ministry of Environment, Lands and Parks Wildlife Branch, Victoria, 2001).
30. Stuart-Smith, A. K., Bradshaw, C. J. A., Boutin, S., Hebert, D. M. & Rippin, A. B. Woodland Caribou Relative to Landscape Patterns in Northeastern Alberta. *J Wildl Manage* **61**, 622 (1997).
31. Wilson, K. S., Pond, B. A., Brown, G. S. & Schaefer, J. A. The biogeography of home range size of woodland caribou *Rangifer tarandus caribou*. *Divers Distrib* **25**, 205–216 (2019).
32. Wood, M. D. Seasonal habitat use and movements of woodland caribou in the Omineca Mountains, north central British Columbia, 1991-1993. *Rangifer* **16**, 365 (1996).
33. Parker, G. R. Biology of the Kaminuriak Population of barren-ground caribou. in *Canadian Wildlife Service Report Series* vol. 20 (1972).
34. COSEWIC. *COSEWIC Assessment and Status Report on the Caribou Rangifer Tarandus, Barren-Ground Population, in Canada*. (2016).
35. Joly, K. *et al.* Longest terrestrial migrations and movements around the world. *Sci Rep* **9**, 15333 (2019).
36. Berger, J. The Last Mile: How to Sustain Long-Distance Migration in Mammals. *Conserv Biol* **18**, 320–331 (2004).
37. Bergerud, A. T. & Luttich, S. N. Predation risk and optimal foraging trade-off in the demography and spacing of the George River Herd, 1958 to 1993. *Rangifer* **23**, 169 (2003).
38. Schaefer, J. A., Bergman, C. M. & Luttich, S. N. Site fidelity of female caribou at multiple spatial scales. *Landsc Ecol* **15**, 731–739 (2000).
39. Joly, K., Gurarie, E., Hansen, D. A. & Cameron, M. D. Seasonal patterns of spatial fidelity and temporal consistency in the distribution and movements of a migratory ungulate. *Ecol Evol* **11**, 8183–8200 (2021).

40. Meldgaard, M. *The Greenland Caribou - Zoogeography, Taxonomy and Population Dynamics*. *ARCTIC* vol. 20 (Meddelelser om Grønland, Copenhagen, 1986).
41. Joly, K., Jandt, R. R., Meyers, C. R. & Cole, M. J. Changes in vegetative cover on Western Arctic Herd winter range from 1981 to 2005: potential effects of grazing and climate change. *Rangifer* **27**, 199 (2007).
42. Bergerud, A. T. & Mercer, W. E. Caribou Introductions in Eastern North America. *Wildl Soc Bull* **17**, 111–120 (1989).
43. Burch, Jr. E. S. The Caribou/Wild Reindeer as a Human Resource. *Am Antiq* **37**, 339–368 (1972).
44. Murie, O. J. Alaska-Yukon caribou. in *North American Faunas of the Biological Survey 1935-1938* vol. 54 95–415 (United States Department of Agriculture, Bureau of Biological Survey, Washington, D. C., 1953).
45. Whitten, K. R., Mauer, F. J., Garner, G. W. & Russell, D. E. Fall and winter movements and distribution, and annual mortality patterns of the Porcupine caribou herd, 1983-1984. in *Arctic National Wildlife Refuge coastal plain resource assessment, 1984 update report, baseline study of the fish, wildlife and their habitats* (eds. Garner, G. W. & Reynolds, P. E.) 515–526 (United States Fish and Wildlife Service, Anchorage, 1985).
46. Nicholson, K. L., Arthur, S. M., Horne, J. S., Garton, E. O. & Del Vecchio, P. A. Modeling Caribou Movements: Seasonal Ranges and Migration Routes of the Central Arctic Herd. *PLoS One* **11**, e0150333 (2016).
47. Johnson, C. J., Parker, K. L., Heard, D. C. & Gillingham, M. P. A Multiscale Behavioral Approach to Understanding the Movements of Woodland Caribou. *Ecol Appl* **12**, 1840 (2002).
48. COSEWIC. *COSEWIC Assessment and Status Report on the Plains Bison Bison Bison Bison in Canada*. (2004).
49. Brown, G. S., Mallory, F. F. & Rettie, W. J. Range size and seasonal movement for female woodland caribou in the boreal forest of northeastern Ontario. *Rangifer* **14**, 227–233 (2003).
50. Cameron, R. D. & Whitten K. R. Seasonal Movements and Sexual Segregation of Caribou Determined by Aerial Survey. *J Wildl Manage* **43**, 626–633 (1979).
51. Duquette, L. S. & Klein, D. R. Activity budgets and group size of caribou during spring migration. *Can J Zool* **65**, 164–168 (1987).
52. Edmonds, E. J. Population status, distribution, and movements of woodland caribou in west central Alberta. *Can J Zool* **66**, 817–826 (1988).
53. Heard, D. C. & Vagt, K. L. Caribou in British Columbia: A 1996 status report. *Rangifer* **18**, 117 (1998).
54. Hansen, R. M. Foods of Free-Roaming Horses in Southern New Mexico. *J Range Manag* **29**, 347 (1976).
55. Hubbard, R. E. & Hansen, R. M. Diets of wild horses, cattle, and mule deer in the Piceance Basin, Colorado. *J Range Manag* **29**, 389–392 (1976).
56. Salter, R. E. & Hudson, R. J. Social organization of feral horses in western Canada. *Appl Anim Ethol* **8**, 207–223 (1982).
57. McInnis, M. L. & Vavra, M. Dietary Relationships among Feral Horses, Cattle, and Pronghorn in Southeastern Oregon. *J Range Manag* **40**, 60–66 (1967).
58. Krysl, L. J. *et al.* Horses and Cattle Grazing in the Wyoming Red Desert, I. Food Habits and Dietary Overlap. *J Range Manag* **37**, 72 (1984).
59. Crane, K. K., Smith, M. A. & Reynolds, D. Habitat Selection Patterns of Feral Horses in Southcentral Wyoming. *J Range Managt* **50**, 374 (1997).

60. Schulz, E. & Kaiser, T. M. Historical distribution, habitat requirements and feeding ecology of the genus *Equus* (Perissodactyla). *Mamm Rev* **43**, 111–123 (2013).
61. Xu, W. *et al.* Seasonal diet of Khulan (Equidae) in Northern Xinjiang, China. *Ital J Zool* **79**, 92–99 (2012).
62. Crowell-Davis, S. L., Houpt, K. A. & Carnevale, J. Feeding and Drinking Behavior of Mares and Foals with Free Access to Pasture and Water. *J Anim Sci* **60**, 883–889 (1985).
63. Scheibe, K. M., Eichhorn, K., Kalz, B., Streich, W. J. & Scheibe, A. Water consumption and watering behavior of Przewalski horses (*Equus ferus przewalskii*) in a semireserve. *Zoo Biol* **17**, 181–192 (1998).
64. Schoenecker, K. A., King, S. R. B., Nordquist, M. K., Nandintsetseg, D. & Cao, Q. Habitat and Diet of Equids. In *Wild Equids: Ecology, Management, and Conservation* (eds. Ransom, J. I. & Kaczensky, P.) 41–57 (John Hopkins University Press, Baltimore, 2016).
65. Salter, R. E. & Hudson, R. J. Feeding Ecology of Feral Horses in Western Alberta. *J Range Manag* **32**, 221 (1979).
66. Mejdell, C. M. & Bøe, K. E. Responses to climatic variables of horses housed outdoors under Nordic winter conditions. *Can J Anim Sci* **85**, 307–308 (2005).
67. Feh, C., Shah, N., Rowen, M., Reading, R. & Goyal, S. P. Status and action plan for the Asiatic Wild Ass (*Equus hemionus*). in *Equids: Zebras, Asses and Horses. Status survey and conservation action plan* (ed. Moehlman, P.) 62–70 (International Union for Conservation of Nature, Gland, 2002).
68. Kaczensky, P., Dresley, V., Vetter, D., Otgonbayar, H. & Walzer, C. Water use of Asiatic wild asses in the Mongolian Gobi. *Exploration into the Biological Resources of Mongolia* **11**, 291–298 (2010).
69. Boyd, L. & Keiper, R. Behavioural ecology of feral horses. in *The Domestic Horse, The Evolution, Development and Management of Its Behaviour* (eds. Mills, D. S. & McDonnell, S. M.) 55–82 (Cambridge University Press, Cambridge, 2005).
70. Petersen, S. L., Scasta, J. D., Schoenecker, K. A. & Hennig, J. D. Feral Equids. Iin *Rangeland Wildlife Ecology and Conservation* (eds. McNew, L. B., Dahlgren, D. K. & Beck, J. L.) 735–757 (Springer International Publishing, Cham, 2023). doi:10.1007/978-3-031-34037-6_21.
71. Girard, T. L., Bork, E. W., Nielsen, S. E. & Alexander, M. J. Seasonal Variation in Habitat Selection by Free-Ranging Feral Horses Within Alberta's Forest Reserve. *Rangel Ecol Manag* **66**, 428–437 (2013).
72. Bahloul, K. *et al.* Social organization and dispersion of introduced kulans (*Equus hemionus kulan*) and Przewalski horses (*Equus przewalski*) in the Bukhara Reserve, Uzbekistan. *J Arid Environ* **47**, 309–323 (2001).
73. King, S. R. B. & Gurnell, J. Habitat use and spatial dynamics of takhi introduced to Hustai National Park, Mongolia. *Biol Conserv* **124**, 277–290 (2005).
74. St-Louis, A. & Côté, S. D. *Equus Kiang* (Perissodactyla: Equidae). *Mamm Species* **835**, 1–11 (2009).
75. Schoenecker, K. A., Esmaeili, S. & King, S. R. B. Seasonal resource selection and movement ecology of free-ranging horses in the western United States. *J Wildl Manage* **87**, (2023).
76. Ganskopp, D. & Vavra, M. Habitat Use by Feral Horses in the Northern Sagebrush Steppe. *Journal of Range Management* **39**, 207 (1986).

77. King, S. R. B. Home range and habitat use of free-ranging Przewalski horses at Hustai National Park, Mongolia. *Appl Anim Behav Sci* **78**, 103–113 (2002).
78. King, S. R. B. & Gurnell, J. Habitat use and spatial dynamics of takhi introduced to Hustai National Park, Mongolia. *Biol Conserv* **124**, 277–290 (2005).
79. Linklater, W. L., Cameron, E. Z., Stafford, K. J. & Veltman, C. J. Social and spatial structure and range use by Kaimanawa wild horses (*Equus caballus*: Equidae). *N Z J Ecol* **24**, 139–152 (2000).
80. Lugauer, B. Differences in movement pattern between Asiatic wild ass (*Equus hemionus)* and Przewalski's horse (*Equus ferus prezewalskii*)*.* (University of Vienna, Vienna, 2010).
81. Hennig, J. D., Beck, J. L. & Scasta, J. D. Spatial ecology observations from feral horses equipped with global positioning system transmitters. *Hum Wildl Interact* **12**, 75–84 (2018).
82. Berger, J. *Wild Horses of the Great Basin: Social Competition and Population Size*. (University of Chicago Press, Chicago, 1986).
83. Kaczensky, P., Ganbaatar, O., Von Wehrden, H. & Walzer, C. Resource selection by sympatric wild equids in the Mongolian Gobi. *J Appl Ecol* **45**, 1762–1769 (2008).
84. Linklater, W. L. Adaptive explanation in socio-ecology: lessons from the Equidae. *Biol Rev Camb Philos Soc* **75**, (2000).
85. Klingel, H. Social organization of feral horses. *J Reprod Fertil Suppl* **32**, 89–95 (1982).
86. Keiper, R. R. & Sambraus, H. H. The stability of equine dominance hierarchies and the effects of kinship, proximity and foaling status on hierarchy rank. *Appl Anim Behav Sci* **16**, 121–130 (1986).
87. Ransom, J. I. *et al.* Wild and Feral Equid Population Dynamics. in *Wild Equids: Ecology, Management, and Conservation* (eds. Ransom, J. I. & Kaczensky, P.) 68–86 (John Hopkins University Press, Baltimore, 2016).
88. Boyd, L., Scroll, A., Nowzari, H. & Bouskila, A. Social Organization of Wild Equids. in *Wild Equids: Ecology, Management, and Conservation* (eds. Ransom, J. I. & Kaczensky, P.) 7–22 (John Hopkins University Press, Baltimore, 2016).
89. Moss, C. J. The demography of an African elephant (*Loxodonta africana*) population in Amboseli, Kenya. *J Zool* **255**, 145–156 (2001).
90. Sukumar, R. *The Living Elephants: Evolutionary Ecology, Behavior, and Conservation*. (University of Oxford Press, New York, 2003).
91. Vancuylenberg, B. W. B. Feeding behaviour of the asiatic elephant in South-East Sri Lanka in relation to conservation. *Biol Conserv* **12**, 33–54 (1977).
92. Blake, S. The Ecology of Forest Elephant Distribution and its Implications for Conservation. (University of Edinburgh, Edinburgh, 2002).
93. Tchamba, M. N. & Seme, P. M. Diet and feeding behaviour of the forest elephant in the Santchou Reserve, Cameroon. *Afr J Ecol* **31**, 165–171 (1993).
94. Douglas-Hamilton, I. On the ecology and behaviour of the African elephant: the elephants of Lake Manyara. (University of Oxford, Oxford, 1972).
95. White, L. J. T., Tutin, C. E. G. & Fernandez, M. Group composition and diet of forest elephants, *Loxodonta africana cyclotis* Matschie 1900, in the Lopé Reserve, Gabon. *Afr J Ecol* **31**, 181–199 (1993).

96. Ahrestani, F. S., Heitkönig, I. M. A. & Prins, H. H. T. Diet and habitat-niche relationships within an assemblage of large herbivores in a seasonal tropical forest. *J Trop Ecol* **28**, 385–394 (2012).
97. Codron, J. *et al.* Landscape-scale feeding patterns of African elephant inferred from carbon isotope analysis of feces. *Oecologia* **165**, 89–99 (2011).
98. Hansen, R. M., Mugambi, M. M. & Bauni, S. M. Diets and Trophic Ranking of Ungulates of the Northern Serengeti. *J Wildl Manage* **49**, 823 (1985).
99. Owen-Smith, N. & Chafota, J. Selective feeding by a megaherbivore, the African elephant (*Loxodonta africana*). *J Mammal* **93**, 698–705 (2012).
100. De Boer, W. F., Ntumi, C. P., Correia, A. U. & Mafuca, J. M. Diet and distribution of elephant in the Maputo Elephant Reserve, Mozambique. *Afr J Ecol* **38**, 188–201 (2000).
101. Sukumar, R. *The Asian Elephant: Ecology and Management*. (Cambridge University Press, Cambridge, 1989).
102. Codron, J. *et al.* Elephant (*Loxodonta Africana*) diets in Kruger National Park, South Africa: Spatial and Landscape differences. *J Mammal* **87**, 27–34 (2006).
103. Koch, P. L., Fisher, D. C. & Dettman, D. Oxygen isotope variation in the tusks of extinct proboscideans: A measure of season of death and seasonality. *Geology* **17**, 515 (1989).
104. Ayliffe, L. K., Lister, A. M. & Chivas, A. R. The preservation of glacial-interglacial climatic signatures in the oxygen isotopes of elephant skeletal phosphate. *Palaeogeogr Palaeoclimatol Palaeoecol* **99**, 179–191 (1992).
105. Alfred, R. *et al.* Home Range and Ranging Behaviour of Bornean Elephant (*Elephas maximus borneensis*) Females. *PLoS One* **7**, e31400 (2012).
106. Haynes, G. *Mammoths, Mastodonts, and Elephants. Biology, Behaviour, and the Fossil Record*. (Cambridge University Press, Cambridge, 1991).
107. Poole, J. The African Elephant. in *Studying Elephants* (ed. Kangwana, K.) 1–9 (The African Wildlife Foundation, Nairobi, 1996).
108. Shannon, G., Page, B., Slotow, R. & Duffy, K. J. African elephant home range and habitat selection in Pongola Game Reserve, South Africa. *Afr Zool* **41**, 37–44 (2006).
109. Sukumar, R. A brief review of the status, distribution and biology of wild Asian elephants Elephas maximus. *International Zoo Yearbook* **40**, 1–8 (2006).
110. Babassa, D. Habitat selection by elephants in Bwindi Impenetrable National Park, south-western Uganda. *Afr J Ecol* **38**, 116–122 (2000).
111. De Boer, W. F., Ntumi, C. P., Correia, A. U. & Mafuca, J. M. Diet and distribution of elephant in the Maputo Elephant Reserve, Mozambique. *Afr J Ecol* **38**, 188–201 (2000).
112. de Knegt, H. J. *et al.* The spatial scaling of habitat selection by African elephants. *J Anim Ecol* **80**, 270–281 (2011).
113. Duffy, K. J., Dai, X., Shannon, G., Slotow, R. & Page, B. Movement patterns of African elephants (*Loxodonta africana*) in different habitat types. *South African Journal of Wildlife Research* **41**, 21–28 (2011).
114. Field, C. R. Elephant Ecology in the Queen Elizabeth National Park, Uganda. *Afr J Ecol* **9**, 99–123 (1971).
115. Koirala, R. K., Ji, W., Aryal, A., Rothman, J. & Raubenheimer, D. Dispersal and ranging patterns of the Asian Elephant (*Elephas maximus*) in relation to their interactions with humans in Nepal. *Ethol Ecol Evol* 1–12 (2015)
116. Stokke, S. & Du Toit, J. T. Sexual segregation in habitat use by elephants in Chobe National Park, Botswana. *Afr J Ecol* **40**, 360–371 (2002).

117. Ngene, S. *et al.* Home range sizes and space use of African elephants (*Loxodonta africana*) in the Southern Kenya and Northern Tanzania borderland landscape. *Int J Biodivers Conserv* **9**, 9–26 (2017).
118. Leggett, K. E. A. Home range and seasonal movement of elephants in the Kunene Region, northwestern Namibia. *Afr Zool* **41**, 17–36 (2006).
119. Leuthold, W. Spatial organization and strategy of habitat utilization of elephants in Tsavo National Park, Kenya. *Zeitschrift für Saugertierkunde* **42**, 358–397 (1977).
120. Leuthold, W. & Sale, J. B. Movements and patterns of habitat utilization of elephants in Tsavo National Park, Kenya. *Afr J Ecol* **11**, 369–384 (1973).
121. Purdon, A., Mole, M. A., Chase, M. J. & van Aarde, R. J. Partial migration in savanna elephant populations distributed across southern Africa. *Sci Rep* **8**, 11331 (2018).
122. Tshipa, A. *et al.* Partial migration links local surface-water management to large-scale elephant conservation in the world's largest transfrontier conservation area. *Biol Conserv* **215**, 46–50 (2017).
123. Moss, C. J. & Poole, J. H. Relationships and social structure in African elephants. Iin *Primate Social Relationships: an Integrated Approach* (ed. Hinde, R. A.) 315–325 (Blackwell Scientific Publications, Oxford, 1983).
124. Vidya, T. N. C. & Sukumar. R. Social and reproductive behaviour in elephants. *Curr Sci* **89**, 1200–1207 (2005).
125. Poole, J. H. Rutting Behavior in African Elephants: the Phenomenon of Musth. *Behaviour* **102**, 283–316 (1987).
126. LaDue, C. A., Vandercone, R. P. G., Kiso, W. K. & Freeman, E. W. Social Behavior and Group Formation in Male Asian Elephants (*Elephas maximus*): The Effects of Age and Musth in Wild and Zoo-Housed Animals. *Animals* **12**, 1215 (2022).
127. Capo, R. C., Stewart, B. W. & Chadwick, O. A. Strontium isotopes as tracers of ecosystem processes: theory and methods. *Geoderma* **82**, 197–225 (1998).
128. Montgomery, J. Passports from the past: Investigating human dispersals using strontium isotope analysis of tooth enamel. *Ann Hum Biol* **37**, 325–346 (2010).
129. Price, T. D., Burton, J. H. & Bentley, R. A. The characterization of biologically available strontium isotope ratios for the study of prehistoric migration. *Archaeometry* **44**, 117–135 (2002).
130. Bentley, R. A. Strontium isotopes from the earth to the archaeological skeleton: A review. *J Archaeol Method Theory* **13**, 135–187 (2006).
131. Green, G. P., Bestland, E. A. & Walker, G. S. Distinguishing sources of base cations in irrigated and natural soils: evidence from strontium isotopes. *Biogeochemistry* **68**, 199–225 (2004).
132. Vitousek, P. M., Kennedy, M. J., Derry, L. A. & Chadwick, O. A. Weathering versus atmospheric sources of strontium in ecosystems on young volcanic soils. *Oecologia* **121**, 255–259 (1999).
133. McConnell, D. *Apatite, Its Crystal Chemistry, Mineralogy, Utilization and Geologic and Biologic Occurrences*. (Springer Vienna, Vienna, 1973).
134. Britton, K., Grimes, V., Dau, J. & Richards, M. P. Reconstructing faunal migrations using intra-tooth sampling and strontium and oxygen isotope analyses: a case study of modern caribou (*Rangifer tarandus granti*). *J Archaeol Sci* **36**, 1163–1172 (2009).
135. Julien, M.-A. *et al.* Steppe Bison Paleobiology through the Scope of Stable Isotopes and Zooarchaeology. *Geophys Res Abstr* **12**, 1–2 (2010).

136. Pellegrini, M. *et al.* Faunal migration in late-glacial central Italy: Implications for human resource exploitation. *Rapid Comm Mass Spectrom* **22**, 1714–1726 (2008).
137. Slovak, N. M. & Paytan, A. Applications of Sr Isotopes in Archaeology. in *Adv Isotope Geochem* 743–768 (Springer, 2012).
138. Willmes, M. *et al.* Mapping of bioavailable strontium isotope ratios in France for archaeological provenance studies. *Applied Geochemistry* **90**, 75–86 (2018).
139. Funck, J., Bataille, C., Rasic, J. & Wooller, M. A bio-available strontium isoscape for eastern Beringia: a tool for tracking landscape use of Pleistocene megafauna. *J Quat Sci* **36**, 76–90 (2021).
140. Crowley, B. E., Miller, J. H. & Bataille, C. P. Strontium isotopes (87Sr/86Sr) in terrestrial ecological and palaeoecological research: empirical efforts and recent advances in continental-scale models. *Biol Rev* **92**, 43–59 (2017).
141. Bataille, C. P. *et al.* A bioavailable strontium isoscape for Western Europe: A machine learning approach. *PLoS One* **13**, 1–27 (2018).
142. Snoeck, C. *et al.* Towards a biologically available strontium isotope baseline for Ireland. *Sci Total Environ* **712**, 136248 (2020).
143. Barakat, S. *et al.* Laser ablation strontium isotopes and spatial assignment show seasonal mobility in red deer (*Cervus elaphus*) at Lazaret Cave, France (MIS 6). *Front Ecol Evol* **11**, 1–14 (2023).
144. Britton, K. *et al.* Multi-isotope zooarchaeological investigations at Abri du Maras: The paleoecological and paleoenvironmental context of Neanderthal subsistence strategies in the Rhône Valley during MIS 3. *J Hum Evol* **174**, 103292 (2023).
145. Britton, K. *et al.* Strontium isotope evidence for migration in late Pleistocene *Rangifer*: Implications for Neanderthal hunting strategies at the Middle Palaeolithic site of Jonzac, France. *J Hum Evol* **61**, 176–185 (2011).
146. Maurer, A. F. *et al.* Bioavailable $^{87}Sr/^{86}Sr$ in different environmental samples - Effects of anthropogenic contamination and implications for isoscapes in past migration studies. *Sci Total Environ* **433**, 216–229 (2012).
147. Sillen, A., Hall, G., Richardson, S. & Armstrong, R. $^{87}Sr/^{86}Sr$ ratios in modern and fossil food-webs of the Sterkfontein Valley: implications for early hominid habitat preference. *Geochim Cosmochim Acta* **62**, 2463–2473 (1998).
148. Britton, K. *et al.* Sampling Plants and Malacofauna in $^{87}Sr/^{86}Sr$ Bioavailability Studies: Implications for Isoscape Mapping and Reconstructing of Past Mobility Patterns. *Front Ecol Evol* **8**, (2020).
149. Dansgaard, W. Stable isotopes in precipitation. *Tellus* 436–468 (1964).
150. Rozanski, K., Araguás-Araguás, L. & Gonfiantini, R. Isotopic Patterns in Modern Global Precipitation. Iin *Climate Change in Continental Isotopic Records* 1–36 (American Geophysical Union, 2013).
151. Bowen, G. J. & Revenaugh, J. Interpolating the isotopic composition of modern meteoric precipitation. *Water Resour Res* **39**, (2003).
152. Bowen, G. J. Spatial analysis of the intra-annual variation of precipitation isotope ratios and its climatological corollaries. *J Geophys Res: Atmospheres* **113**, 1–10 (2008).
153. Fricke, H. C. & O'Neil, J. R. Inter- and intra-tooth variation in the oxygen isotope composition of mammalian tooth enamel phosphate: Implications for palaeoclimatological and palaeobiological research. *Palaeogeogr Palaeoclimatol Palaeoecol* **126**, 91–99 (1996).

154. Pederzani, S. & Britton, K. Oxygen isotopes in bioarchaeology: Principles and applications, challenges and opportunities. *Earth Sci Rev* **188**, 77–107 (2019).
155. Rozanski, K. Deuterium and oxygen-18 in European groundwaters — Links to atmospheric circulation in the past. *Chem Geol* **52**, 349–363 (1985).
156. Gat, J. R. Stable Isotopes of Fresh and Saline Lakes. Iin *Physics and Chemistry of Lakes* 139–165 (Springer Berlin Heidelberg, Berlin, Heidelberg, 1995)..
157. Gibson, J. J. & Reid, R. Stable isotope fingerprint of open-water evaporation losses and effective drainage area fluctuations in a subarctic shield watershed. *J Hydrol (Amst)* **381**, 142–150 (2010).
158. Krabbenhoft, D. P., Bowser, C. J., Anderson, M. P. & Valley, J. W. Estimating groundwater exchange with lakes: 1. The stable isotope mass balance method. *Water Resour Res* **26**, 2445–2453 (1990).
159. Bowen, G. J., Kennedy, C. D., Liu, Z. & Stalker, J. Water balance model for mean annual hydrogen and oxygen isotope distributions in surface waters of the contiguous United States. *J Geophys Res* **116**, G04011 (2011).
160. Jeelani, G., Shah, R. A., Jacob, N. & Deshpande, R. D. Estimation of snow and glacier melt contribution to Liddar stream in a mountainous catchment, western Himalaya: an isotopic approach. *Isotopes Environ Health Stud* **53**, 18–35 (2017).
161. Seiler, K.-P. & Gat, J. R. Mechanisms and Processes of Recharge. in 31–68 (2007).
162. Darling, W. G., Bath, A. H. & Talbot, J. C. The O and H stable isotope composition of freshwaters in the British Isles. 2. Surface waters and groundwater. *Hydrol Earth Syst Sci* **7**, 183–195 (2003).
163. Congjian, S., Yaning, C., Weihong, L., Xingong, L. & Yuhui, Y. Isotopic time series partitioning of streamflow components under regional climate change in the Urumqi River, northwest China. *Hydrological Sciences Journal* **61**, 1443–1459 (2016).
164. Rank, D., Wyhlidal, S., Schott, K., Weigand, S. & Oblin, A. Temporal and spatial distribution of isotopes in river water in Central Europe: 50 years experience with the Austrian network of isotopes in rivers. *Isotopes Environ Health Stud* **54**, 115–136 (2018).
165. Levin, N. E., Cerling, T. E., Passey, B. H., Harris, J. M. & Ehleringer, J. R. A stable isotope aridity index for terrestrial environments. *Proceedings of the National Academy of Sciences* **103**, 11201–11205 (2006).
166. Flanagan, L. B. & Ehleringer, J. R. Stable Isotope Composition of Stem and Leaf Water: Applications to the Study of Plant Water Use. *Funct Ecol* **5**, 270 (1991).
167. Cernusak, L. A. *et al.* Stable isotopes in leaf water of terrestrial plants. *Plant Cell Environ* **39**, 1087–1102 (2016).
168. Bryant, J. D. & Froelich, P. N. A model of oxygen isotope fractionation in body water of large mammals. *Geochim Cosmochim Acta* **59**, 4523–4537 (1995).
169. Longinelli, A. Oxygen isotopes in mammal bone phosphate: A new tool for paleohydrological and paleoclimatological research? *Geochlmrca d Cosmochimrca Acla* **48**, 385–390 (1984).
170. Bryant, J. D., Luz, B. & Froelich, P. N. Oxygen isotopic composition of fossil horse tooth phosphate as a record of continental paleoclimate. *Palaeogeogr Palaeoclimatol Palaeoecol* **107**, 303–316 (1994).

171. Cain, J. W., Krausman, P. R., Rosenstock, S. S. & Turner, J. C. Mechanisms of Thermoregulation and Water Balance in Desert Ungulates. *Wildl Soc Bull* **34**, 570–581 (2006).
172. Brown, G. & Lynch, J. Some aspects of the water balance of sheep at pasture when deprived of drinking water. *Aust J Agric Res* **23**, 669 (1972).
173. Balasse, M. Potential biases in sampling design and interpretation of intra-tooth isotope analysis. *Int J Osteoarchaeol* **13**, 3–10 (2003).
174. Balasse, M., Obein, G., Ughetto-Monfrin, J. & Mainland, I. Investigating seasonality and season of birth in past herds: A reference set of sheep enamel stable oxygen ^{isotope} ratios. *Archaeometry* **54**, 349–368 (2012).
175. Bernard, A. *et al.* Pleistocene seasonal temperature variations recorded in the δ18O of Bison priscus teeth. *Earth Planet Sci Lett* **283**, 133–143 (2009).
176. Blumenthal, S. A., Cerling, T. E., Smiley, T. M., Badgley, C. E. & Plummer, T. W. Isotopic records of climate seasonality in equid teeth. *Geochim Cosmochim Acta* **260**, 329–348 (2019).
177. Richards, M. P. *et al.* Temporal variations in Equus tooth isotope values (C,N,O) from the Middle Paleolithic site of Combe Grenal, France (ca. 150,000 to 50,000 BP). *J Archaeol Sci Rep* **14**, 189–198 (2017).
178. Price, T. D., Meiggs, D., Weber, M. J. & Pike-Tay, A. The migration of Late Pleistocene reindeer: isotopic evidence from northern Europe. *Archaeol Anthropol Sci* **9**, 371–394 (2017).
179. Tornero, C. *et al.* The altitudinal mobility of wild sheep at the Epigravettian site of Kalavan 1 (Lesser Caucasus, Armenia): Evidence from a sequential isotopic analysis in tooth enamel. *J Hum Evol* **97**, 27–36 (2016).
180. Tornero, C. *et al.* Vertical sheep mobility along the altitudinal gradient through stable isotope analyses in tooth molar bioapatite, meteoric water and pastures: A reference from the Ebro valley to the Central Pyrenees. *Quaternary International* **484**, 94–106 (2018).
181. Gigleux, C., Grimes, V., Tütken, T., Knecht, R. & Britton, K. Reconstructing caribou seasonal biogeography in Little Ice Age (late Holocene) Western Alaska using intra-tooth strontium and oxygen isotope analysis. *J Archaeol Sci Rep* **23**, 1043–1054 (2019).
182. Julien, M. A. *et al.* Were European steppe bison migratory? ^{18}O, ^{13}C and Sr intra-tooth isotopic variations applied to a palaeoethological reconstruction. *Quat Int* **271**, 106–119 (2012).
183. Bocherens, H. Isotopic biogeochemistry and the palaeoecology of the mammoth steppe fauna. *Deinsea* **9**, 57–76 (2003).
184. Ehleringer, J. R., Rundel, P. W. & Nagy, K. A. Stable isotopes in physiological ecology and food web research. *Trends Ecol Evol* **1**, 42–45 (1986).
185. Smith, B. N., Oliver, J. & McMillan, C. Influence of carbon source, oxygen concentration, light intensity, and temperature on $^{13}C/^{12}C$ ratios in plant tissues. *Botanical Gazette* **137**, 99–104 (1976).
186. Farquhar, G., O'Leary, M. & Berry, J. On the Relationship Between Carbon Isotope Discrimination and the Intercellular Carbon Dioxide Concentration in Leaves. *Functional Plant Biology* **9**, 121 (1982).
187. Tieszen, L. L. Natural Variations in the Carbon Isotope Values of Plants: Implications for Archaeology, Ecology, and Paleoecology. *J Archaeol Sci* **18**, 227-248 (1991).

188. Kohn, M. J. Carbon isotope compositions of terrestrial C3 plants as indicators of (paleo)ecology and (paleo)climate. *Proc Natl Acad Sci USA* **107**, 19691–19695 (2010).
189. Balesdent, J., Girardin, C. & Mariotti, A. Site-related ^{13}C of tree leaves and soil organic matter in a temperate forest. *Ecology* **74**, 1713–1721 (1993).
190. Bonafini, M., Pellegrini, M., Ditchfield, P. & Pollard, A. M. Investigation of the 'canopy effect' in the isotope ecology of temperate woodlands. *J Archaeol Sci* **40**, 3926–3935 (2013).
191. Fizet, M. *et al.* Effect of diet, physiology and climate on carbon and nitrogen stable isotopes of collagen in a late pleistocene anthropic palaeoecosystem: Marillac, Charente, France. *J Archaeol Sci* **22**, 67–79 (1995).
192. Heaton, T. H. E. Spatial, Species, and Temporal Variations in the 13 C/ 12 C Ratios of C3 Plants: Implications for Palaeodiet Studies. *J Archaeol Sci* **26**, 637-649 (1999).
193. Cerling, T. E. & Harris, J. M. Carbon isotope fractionation between diet and bioapatite in ungulate mammals and implications for ecological and paleoecological studies. *Oecologia* **120**, 347–363 (1999).
194. DeNiro, M. & Epstein, S. Influence of the Diet on the Distribution of Carbon Isotopes in Animals. *Gecchim Cosmochim Acta* **42**, 495–506 (1978).
195. Vogel, J. C. Isotopic Assessment of the Dietary Habits of Ungulates. *S Afr J Sci* **74**, 298–301 (1978).
196. Drucker, D. G., Bridault, A., Hobson, K. A., Szuma, E. & Bocherens, H. Can carbon-13 in large herbivores reflect the canopy effect in temperate and boreal ecosystems? Evidence from modern and ancient ungulates. *Palaeogeogr Palaeoclimatol Palaeoecol* **266**, 69–82 (2008).
197. Stevens, R. E., Lister, A. M. & Hedges, R. E. M. Predicting diet, trophic level and palaeoecology from bone stable isotope analysis: A comparative study of five red deer populations. *Oecologia* **149**, 12–21 (2006).
198. Drucker, D. G., Bocherens, H. & Billiou, D. Evidence for shifting environmental conditions in Southwestern France from 33 000 to 15 000 years ago derived from carbon-13 and nitrogen-15 natural abundances in collagen of large herbivores. *Earth Planet Sci Lett* **216**, 163–173 (2003).
199. Drucker, D. G. The Isotopic Ecology of the Mammoth Steppe. *Annu Rev Earth Planet Sci* **50**, 395–418 (2022).
200. Passey, B. H. *et al.* Carbon isotope fractionation between diet, breath CO2, and bioapatite in different mammals. *J Archaeol Sci* **32**, 1459–1470 (2005).
201. Drucker, D. G. & Bocherens, H. Carbon stable isotopes of mammal bones as tracers of canopy development and habitat use in temperate and boreal contexts. *Forest Canopies: Forest Production, Ecosystem Health and Climate Conditions* 103–109 (2009).
202. van der Merwe, N. J. & Medina, E. The canopy effect, carbon isotope ratios and foodwebs in amazonia. *J Archaeol Sci* **18**, 249–259 (1991).
203. Pryor, A. J. E., Sázelová, S. & Standish, C. Season of Death and Strontium/Oxygen Isotope Data for Seasonal Mobility of Three Reindeer Prey. in *Dolní Vĕstonice II: Chronology, Paleoethnology, Paleoanthropology* (ed. Svoboda, J.) (Published by the Academy of Sciences of the Czech Republic, Institute of Archaeology, Brno, 2016).
204. Hillson, S. *Teeth*. (Cambridge University Press, 2005).

205. Kohn, M. J., Schoeninger, M. J. & Valley, J. W. Herbivore tooth oxygen isotope compositions: Effects of diet and physiology. *Geochim Cosmochim Acta* **60**, 3889–3896 (1996).
206. Koch, P. L., Tuross, N. & Fogel, M. L. The Effects of Sample Treatment and Diagenesis on the Isotopic Integrity of Carbonate in Biogenic Hydroxylapatite. *J Archaeol Sci* **24**, 417–429 (1997).
207. Lee-Thorp, J. A. Preservation of Biogenic Carbon Isotopic Signals in Plio-Pleistocene Bone and Tooth Mineral. in *Biogeochemical Approaches to Paleodietary Analysis* (eds. Ambrose, S. H. & Katzenberg, A. M.) 89–115 (Kluwer Academic/Plenum Publishers, New York, 2000).
208. Hoppe, K. A., Stover, S. M., Pascoe, J. R. & Amundson, R. Tooth enamel biomineralization in extant horses: Implications for isotopic microsampling. *Palaeogeogr Palaeoclimatol Palaeoecol* **206**, 355–365 (2004).
209. Brown, W. A. B. & Chapman, N. G. The dentition of red deer (Cervus elaphus): a scoring scheme to assess age from wear of the permanent molariform teeth. *J Zool* **224**, 519–536 (1991).
210. Brown, W. A. B. & Chapman, N. G. Age assessment of fallow deer (*Dama dama*): from a scoring scheme based on radiographs of developing permanent molariform teeth. *J Zool* **224**, 367–379 (1991).
211. Britton, K. Multi-isotope analysis and the reconstruction of prey species palaeomigrations and palaeoecology. (Durham University, Durham, 2010).
212. Wright, L. E. & Schwarcz, H. P. Stable carbon and oxygen isotopes in human tooth enamel: Identifying breastfeeding and weaning in prehistory. *Am J Phys Anthropol* **106**, 1–18 (1998).
213. Skoog, R. O. Ecology of the caribou (*Rangifer tarandus granti*) in Alaska. (University of California, Berkley, 1968).
214. Laws, R. M. Age criteria for the African elephant, *Loxodonta a. africana*. *East African Wildlife Journal* **41**, 1–37 (1966).
215. Metcalfe, J. Z., Longstaffe, F. J. & Zazula, G. D. Nursing, weaning, and tooth development in woolly mammoths from Old Crow, Yukon, Canada: Implications for Pleistocene extinctions. *Palaeogeogr Palaeoclimatol Palaeoecol* **298**, 257–270 (2010).
216. Bryant, J. D., Froelich, P. N., Showers, W. J. & Genna, B. J. A Tale of Two Quarries: Biologic and Taphonomic Signatures in the Oxygen Isotope Composition of Tooth Enamel Phosphate from Modern and Miocene Equids. *Palaios* **11**, 397 (1996).
217. Soana, S., Gnudi, G. & Bertoni, G. The Teeth of the Horse: Evolution and Anatomo-Morphological and Radiographic Study of Their Development in the Foetus. *Anat Histol Embryol* **28**, 273–280 (1999).
218. Bendrey, R., Vella, D., Zazzo, A., Balasse, M. & Lepetz, S. Exponentially decreasing tooth growth rate in horse teeth: implications for isotopic analyses. *Archaeometry* **57**, 1104–1124 (2015).
219. Ferretti, M. P. Structure and evolution of mammoth molar enamel. *Acta Palaeontol Pol* **48**, 383–396 (2003).
220. Metcalfe, J. Z. & Longstaffe, F. J. Mammoth tooth enamel growth rates inferred from stable isotope analysis and histology. *Quat Res* **77**, 424–432 (2012).
221. Dirks, W., Bromage, T. G. & Agenbroad, L. D. The duration and rate of molar plate formation in Palaeoloxodon cypriotes and Mammuthus columbi from dental histology. *Quat Int* **255**, 79–85 (2012).

222. Uno, K. T. *et al.* Bomb-curve radiocarbon measurement of recent biologic tissues and applications to wildlife forensics and stable isotope (paleo)ecology. *Proc Natl Acad Sci* **110**, 11736–11741 (2013).
223. Uno, K. T. *et al.* Forward and inverse methods for extracting climate and diet information from stable isotope profiles in proboscidean molars. *Quat Int* **557**, 92–109 (2020).
224. Bonhof, W. J. & Pryor, A. J. E. Proboscideans on Parade: A review of the migratory behaviour of elephants, mammoths, and mastodons. *Quat Sci Rev* **277**, (2022).
225. Mania, D. & Toepfer, V. *Königsaue. Gliederung, Ökologie Und Mittelpaläolithische Funde Der Letzten Eiszeit*. (Deutscher Verlag der Wissenschaften, Berlin, 1973).
226. Jöris, O. Zur Chronostratigraphischen Stellung Der Spätmittelpaläolithischen Keilmessergruppen. Der Versuch Einer Kulturgeographischen Abgrenzung Einer Mittelpaläolithischen Formengruppe in Ihrem Europäischen Kontext. *Ber Röm-German Komm* **84**, 49-153 (2004).
227. Grünberg, J. M. Middle Palaeolithic birch-bark pitch Königsaue. *Antiquity* **76**, 15–16 (2002).
228. Mania, D. 125 000 Jahre Klima- und Umweltentwicklung im mittleren Eibe-Saale-Gebiet. *Hercynia N. F.* **32**, 1–97 (1999).
239. Mania, D. Zum Ablauf der Klimazyklen seit der Elstervereisung im Elbe-Saalegebiet. *Praehistoria Thuringica* **2**, 5–21 (1998).
230. Mania, D. Eiszeitarchäologische Forschungsarbeiten in den Tagebauen des Saale-Elbe-Gebietes. *Veröffentlichungen des Museums für Ur- und Frühgeschichte Potsdam 25. (*Landesmuseum für Vorgeschichte, Potsdam, (1991).
231. Picin, A. Short-term occupations at the lakeshore: a technological reassessment of the open–air site Königsaue (Germany). *Quartär* **63**, 7–32 (2016).
232. Grootes, P. M. Thermal diffusion isotopic enrichment and radiocarbon dating beyond 50 000 years BP. (University of Groningen, Groningen, 1977).
233. Hedges, R. E. M., Pettitt, P. B., Bronk Ramsey, C. & Van Klinken, G. J. Radiocarbon Dates of the Oxford AMS system: Archaeometry Datelist 26. *Archaeometry* **40**, 437–455 (1998).
234. Jöris, O. Bifacially backed knives (*Keilmesser*) in the Central European Middle Palaeolithic. in *Axe Age. Acheulian Tool-making from Quarry to Discard* (eds. Goren-Imbar, N. & Sharon, G.) 287–310 (Equinox Publishing, London, 2006).
234. Ruebens, K. Regional behaviour among late Neanderthal groups in Western Europe: A comparative assessment of late Middle Palaeolithic bifacial tool variability. *J Hum Evol* **65**, 341–362 (2013).
236. Wragg-Sykes, R. M. To see a world in a hafted tool: birch pitch composite technology, cognition and memory in Neanderthals. In *Settlement, Society and Cognition in Human Evolution: Landscapes in the Mind* (eds. Coward, F., Hosfield, R. T., Pope, M. & Wenban-Smith, F. F.) 117–137 (Cambridge University Press, Cambridge, 2015).
237. Koller, J., Baumer, U. & Mania, D. High-tech in the middle Palaeolithic: Neandertal-manufactured pitch identified. *Eur J Archaeol* **4**, 385–397 (2001).
238. Bouchard, J. Essai sur le renne et la climatologie du paléolithique moyen et supérieur. (Université de Paris, Paris, 1966).

239. Niklasson, N. Die paläolithische Station bei Der Schneidemühle bei Breitenbach im Kreise Zeitz. *Nachr Deutsche Vorzeit* **3**, 58 (1928).
240. Grünberg, J. M. New AMS Dates for Palaeolithic and Mesolithic Camp Sites and Single Finds in Saxony-Anhalt and Thuringia (Germany). *Proc Prehist Soc* **72**, 95–112 (2006).
241. Schäfer, J. Zur Stratigraphie und Geomorphologie am Aurignacien-Freilandfundplatz Breitenbach-Schlottweh. in *Sonderband 16: Zusammengegraben – Kooperationsprojekte in Sachsen-Anhalt. Tagung vom 17. Bis 20. Mai 2009 im Landesmuseum für Vorgeschichte Halle (Saale)* (ed. Meller, H.) vol. 16 19–26 (Landesmuseum für Vorgeschichte Halle/Saale, Haale/Saale, 2012).
242. Jöris, O. & Moreau, L. Vom Endes des Aurignacian: Zur Chronologischen Stellung des Freilandfundplatzes Breitenbach (Burgenlandkr.) im Kontext des Frühen und Mittleren Jungpaläolithikums in Mitteleuropa. *Archäol Korrespondenzbl* **1**, 1–20 (2010).
243. Moreau, L. Breitenbach-Schneidemühle, Germany: A major Aurignacian open air settlement in Central Europe. *Euras Prehist* **9**, 51–75 (2013).
244. Hahn, J. *Aurignacien, Das Ältere Jungpaläolithikum in Mittel- Und Osteuropa.* (Fundamenta A9, Köln/Wien, 1977).
245. Pohl, G. Die jungpaläolithische Siedlung Breitenbach, Kr. Zeitz, und ihre bisherige Beurteilung. *Jahresschr mitteldeut Vorgesch* **41/42**, 178–190 (1958).
246. Richter, J. Jungpaläolithische Funde aus Breitenbach/Kr. Zeitz im Germanischen Nationalmuseum Nürnberg. *Quartär* **37/38**, 63–96 (1987).
247. Jöris, O., Matthies, T. & Fischer, P. Am Rande der bewohnten Welt. Vom Leben in den trundrenähnlichen Graslandschaften des nördlichen Mitteleuropa vor 34 000 Jahren. In *Klimagewalten – treibende Kraft der Evolution. Begleitband zur Sonderausstellung im Landesmuseum für Vorgeschichte Halle (Saale),* (ed. Meller, H.) 318–331 (Landesamt für Denkmalpflege und Archäologie Sachsen-Anhalt, Halle (Saale), 2017).
248. Moreau, L. & Jöris, O. La fn de l'Aurignacien. Au sujet de la position chronologique de la station de plein air de Breitenbachdans le contexte du Paléolithique supérieur ancien en Europe centrale. *Mémoire LVI de la Société préhistorique française* 365–414 (2011).
249. Jöris, O., Neugebauer-Maresch, C., Weninger, B. & Street, M. The Radiocarbon Chronology of the Aurignacian to Mid-Upper Palaeolithic Transition along the Upper and Middle Danube. In *New Aspects of the Central and Eastern European Upper Palaeolithic. Methods, chronology, technology and subsistence* (eds. Neugebauer-Maresch, C. & Owen, L.) 101–138 (Prehistoric Commission of the Austrian Academy of Sciences, Vienna, 2010).
250. Street, M. & Tergberger, T. The German Upper Palaeolithic 35,000-15,000 BP. New Dates and Insights with Emphasis on the Rhineland. in *Hunters of the Golden Age. The Mid Upper Palaeolithic of Eurasia 30,000-20,000 BP.* (eds. W. Roebroeks, Mussi, M., Svoboda, J. & Fennema, K.) 281–297 (Analecta Praehist. Leidensia, 2000).
251. Niklasson, N. Die Grabung auf der jungpalaolithischen Station bei der Schneidemühle bei Breitenbach, Kreis Zeitz. *Nachrichrenblatt für deutsche Vorzeit* **3**, 58 (1927).
252. Groiß, J. Th. Fossilfunde aus dem Aurignacien von Breitenbach, Kreis Zeitz, Bez. Halle. *Quartär* **37/38**, 97–100 (1987).

253. Matthies, T., Fischer, P. & Jöris, O. Hunted or collected? A critical re-evaluation of the Proboscidean remains at the Aurignacian open-air site Breitenbach-Schneidemühle (Germany). in *Hugo Obermaier Society 59th Annual Meeting* (Aurich, 2017).
254. Matthies, T. The Exploitation of Fur-bearing Mammals during the Late Aurignacian of Central Europe: a Case Study of the Faunal remains from Breitenbach (Saxony-Anhalt), Germany. (University of Southampton , Southampton, 2010).
255. Hufthammer, A. K. Age determination of reindeer (*Rangifer tarandus* L.). *Archaeozoologia* **7**, 33–41 (1995).
256. Pasda, K. *Osteometry, and Osteological Age and Sex Determination of the Sisimiut Reindeer Population (Rangifer tarandus groenlandicus)*. (British Archaeological Reports, Oxford, 2009).
