# Supplementary material for: Multi-isotope reconstruction of Late Pleistocene large-herbivore biogeography and mobility patterns in Central Europe
Source: Commun Biol. 2024 May 14;7:568. doi: 10.1038/s42003-024-06233-2 (PMC11094090; doi:10.1038/s42003-024-06233-2)
Supplement: Supplementary file 2 — Description of Additional Supplementary Files [file 42003_2024_6233_MOESM2_ESM.pdf]

Description of Additional Supplementary Files

File name: Supplementary Data 1

Description: The source data behind Figure 3 and Figure 4 in the paper

File name: Supplementary Data 2

Description: The source data being Figure 3 and Figure 5 in the paper

File name: Supplementary Data 3

Description: The source data for plant $^{87}Sr/^{86}Sr$ values behind Figure 1 in the paper
