# Supplementary material for: Multi-isotope reconstruction of Late Pleistocene large-herbivore biogeography and mobility patterns in Central Europe
Source: Commun Biol. 2024 May 14;7:568. doi: 10.1038/s42003-024-06233-2 (PMC11094090; doi:10.1038/s42003-024-06233-2)
Supplement: Supplementary file 6 — Reporting Summary [file 42003_2024_6233_MOESM6_ESM.pdf]

nature portfolio

Corresponding author(s): Phoebe Heddell-Stevens

Last updated by author(s): 17.04.2024

Reporting Summary

Nature Portfolio wishes to improve the reproducibility of the work that we publish. This form provides structure for consistency and transparency in reporting. For further information on Nature Portfolio policies, see our Editorial Policies and the Editorial Policy Checklist.

Statistics

For all statistical analyses, confirm that the following items are present in the figure legend, table legend, main text, or Methods section.

n/a	Confirmed	
☐	☒	The exact sample size (*n*) for each experimental group/condition, given as a discrete number and unit of measurement
☐	☒	A statement on whether measurements were taken from distinct samples or whether the same sample was measured repeatedly
☐	☒	The statistical test(s) used AND whether they are one- or two-sided<br>*Only common tests should be described solely by name; describe more complex techniques in the Methods section.*
☐	☒	A description of all covariates tested
☐	☒	A description of any assumptions or corrections, such as tests of normality and adjustment for multiple comparisons
☐	☒	A full description of the statistical parameters including central tendency (e.g. means) or other basic estimates (e.g. regression coefficient) AND variation (e.g. standard deviation) or associated estimates of uncertainty (e.g. confidence intervals)
☐	☒	For null hypothesis testing, the test statistic (e.g. *F*, *t*, *r*) with confidence intervals, effect sizes, degrees of freedom and *P* value noted<br>*Give P values as exact values whenever suitable.*
☒	☐	For Bayesian analysis, information on the choice of priors and Markov chain Monte Carlo settings
☒	☐	For hierarchical and complex designs, identification of the appropriate level for tests and full reporting of outcomes
☒	☐	Estimates of effect sizes (e.g. Cohen's *d*, Pearson's *r*), indicating how they were calculated

Our web collection on statistics for biologists contains articles on many of the points above.

Software and code

Policy information about availability of computer code

Data collection	Thermo Scientific and Nu Mass Spectrometry Software.
Data analysis	Microsoft Excel.

For manuscripts utilizing custom algorithms or software that are central to the research but not yet described in published literature, software must be made available to editors and reviewers. We strongly encourage code deposition in a community repository (e.g. GitHub). See the Nature Portfolio guidelines for submitting code & software for further information.

Data

Policy information about availability of data

All manuscripts must include a data availability statement. This statement should provide the following information, where applicable:

- Accession codes, unique identifiers, or web links for publicly available datasets
- A description of any restrictions on data availability
- For clinical datasets or third party data, please ensure that the statement adheres to our policy

The authors confirm that all data generated during this study are included in this published article, Figures 3, 4 and 5, and the Supplementary Data files 1, 2 and 3. All teeth are the property of the State Office for Heritage Preservation and Archaeology of Saxony-Anhalt, Germany and are available (with photographs) on request.

Human research participants

Policy information about studies involving human research participants and Sex and Gender in Research.

Reporting on sex and gender	N/A
Population characteristics	N/A
Recruitment	N/A
Ethics oversight	N/A

Note that full information on the approval of the study protocol must also be provided in the manuscript.

Field-specific reporting

Please select the one below that is the best fit for your research. If you are not sure, read the appropriate sections before making your selection.

☐ Life sciences ☐ Behavioural & social sciences ☒ Ecological, evolutionary & environmental sciences

For a reference copy of the document with all sections, see nature.com/documents/nr-reporting-summary-flat.pdf

Ecological, evolutionary & environmental sciences study design

All studies must disclose on these points even when the disclosure is negative.

Study description	We undertook stable isotope analysis on Late Pleistocene fossil teeth of reindeer, horse and mammoth from two archaeological sites in Central Germany to reconstruct faunal dietary and spatial ecology.
Research sample	We analysed suitably-preserved fossil teeth from the site of Königsaue (n=8) and Breitenbach (n=11), previously excavated from the archaeological sites.
Sampling strategy	As above, faunal fossil teeth was analysed. We sequentially sampled 19 teeth for stable carbon and oxygen isotope and strontium isotope analysis and further bulk sampled 16 teeth for stable carbon and oxygen isotope analysis to investigate seasonality in dietary and spatial ecology.
Data collection	Isotope data was collected by P.H.-S., M.L., E.S. and P.L.R. over a period of two years.
Timing and spatial scale	The whole process of these analyses took a period of two years. The analyses are not time-dependent.
Data exclusions	No data was excluded.
Reproducibility	Identification of faunal material was confirmed by two seperate specialists. All isotopic measurements performed on the Gasbench involve an average of 10 measurements and replicate analysis of an internal enamel standard and external internationally recognised standards. Isotopic measurements performed on the Nu Plasma also incorporate replicate analysis of internal and international standards.
Randomization	N/A
Blinding	N/A

Did the study involve field work? ☐ Yes ☒ No

Reporting for specific materials, systems and methods

We require information from authors about some types of materials, experimental systems and methods used in many studies. Here, indicate whether each material, system or method listed is relevant to your study. If you are not sure if a list item applies to your research, read the appropriate section before selecting a response.

Materials & experimental systems

n/a	Involved in the study
☒	☐ Antibodies
☒	☐ Eukaryotic cell lines
☐	☒ Palaeontology and archaeology
☐	☒ Animals and other organisms
☒	☐ Clinical data
☒	☐ Dual use research of concern

Methods

n/a	Involved in the study
☒	☐ ChIP-seq
☒	☐ Flow cytometry
☒	☐ MRI-based neuroimaging

Palaeontology and Archaeology

Specimen provenance	Königsaue, Saxony-Anhalt, Germany and Breitenbach, Saxony-Anhalt, Germany.
Specimen deposition	The Königsaue material is stored at the State Museum of Prehistory Halle (Saale), Saxony-Anhalt, Germany. The Breitenbach material is currently held at Leibniz-Zentrum für Archäologie (LEIZA), MONREPOS Archaeological Research Centre and Museum for Human Behavioural Evolution, Rhineland-Palatinate, Germany.
Dating methods	No new dates were generated as part of this study.

☒ Tick this box to confirm that the raw and calibrated dates are available in the paper or in Supplementary Information.

Ethics oversight	Deceased Pleistocene reindeer, horse and mammoth were sampled for this study. Additionally modern plant samples collected in the study region were also sampled.

Note that full information on the approval of the study protocol must also be provided in the manuscript.

Animals and other research organisms

Policy information about studies involving animals; ARRIVE guidelines recommended for reporting animal research, and Sex and Gender in Research

Laboratory animals	N/A
Wild animals	N/A
Reporting on sex	N/A
Field-collected samples	N/A
Ethics oversight	Deceased Pleistocene reindeer, horse and mammoth were sampled for this study. Additionally modern plant samples collected in the study region were also sampled.

Note that full information on the approval of the study protocol must also be provided in the manuscript.
